# Construction and validation of a novel angiogenesis pattern to predict prognosis and immunotherapy efficacy in colorectal cancer

**DOI:** 10.18632/aging.205189

**Published:** 2023-11-07

**Authors:** Zhiyong Li, Yang Liu, Peng Guo, Yunwei Wei

**Affiliations:** 1 Department of Emergency Surgery, Peking University People’s Hospital, Xicheng, Beijing 100044, China; 2 Department of Pancreatic and Gastrointestinal Surgery Division, Ningbo Second Hospital, Ningbo, Zhejiang 315010, China; 3 Ningbo Key Laboratory of Intestinal Microecology and Human Major Diseases, Ningbo, Zhejiang 315010, China; 4 Department of Emergency Medicine, Peking University People’s Hospital, Xicheng, Beijing 100044, China; 5 Laboratory of Surgery Oncology, Peking University People’s Hospital, Xicheng, Beijing 100044, China

**Keywords:** colorectal cancer, angiogenesis, prognostic, immunotherapy response, *Fusobacterium nucleatum*, biomarker

## Abstract

Background: Evidence suggests that the tumor microenvironment (TME) affects the tumor active response to immunotherapy. Tumor angiogenesis is closely related to the TME. Nonetheless, the effects of angiogenesis on the TME of colorectal cancer (CRC) remain unknown.

Methods: We comprehensively assessed the angiogenesis patterns in CRC based on 36 angiogenesis-related genes (ARGs). Subsequently, we evaluated the prognostic values and therapeutic sensitivities of angiogenesis patterns using multiple methods. We then performed the machine learning algorithm and functional experiments to identify the prognostic key ARGs. Ultimately, the regulation of gut microbiota on the expression of ARGs was further investigated by using whole genome sequencing.

Results: Two angiogenesis clusters were identified and angiogenesis cluster B was characterized by increased stromal and immunity activation with unfavorable odds of survival. Further, an ARG_score including 9 ARGs to predict recurrence-free survival (RFS) was established and its predominant predictive ability was confirmed. The low ARG_score patients were characterized by a high mutation burden, high microsatellite instability, and immune activation with better prognosis. Moreover, patients with high KLK10 expression were associated with a hot tumor immune microenvironment, poorer immune checkpoint blocking treatment, and shorter survival. The *in vitro* experiments also indicated that *Fusobacterium nucleatum* (*F.n*) infection significantly induced KLK10 expression in CRC.

Conclusions: The quantification of angiogenesis patterns could contribute to predict TME characteristics, prognosis, and individualized immunotherapy strategies. Furthermore, our findings suggest that *F.n* may influence CRC progression through ARGs, which could serve as a clinical biomarker and therapeutic target for *F.n*-infected CRC patients.

## INTRODUCTION

CRC is one of the most common malignancies of the digestive system, and is the second leading cause of cancer-related death worldwide. [1, 2]. The morbidity rate of CRC continues to increase, with nearly 1.8 million individuals diagnosed and over 900,000 deaths each year [3]. Recently, the incidence and mortality of CRC in some European and Northern American countries have decreased. However, CRC incidence and mortality continue to increase in China [4]. Chemotherapeutic regimens for the treatment of advanced CRC (e.g., oxaliplatin and 5-fluorouracil (5-FU)) have made substantial progress [5, 6]. However, the overall results of various targeted drugs currently used for the treatment of advanced CRC (such as the anti-EGFR agent cetuximab and the anti-angiogenesis agent bevacizumab) remain a challenge [7, 8]. Immunotherapy offers additional options and hope for the treatment of CRC patients. In fact, not all types of patients can benefit from immunotherapy [5]. There is therefore an urgent need to construct a novel valuable biomarker that can categorize different features of patients into distinct groups and predict the efficacy of immunotherapy in CRC.

A sufficient supply of oxygen and nutrients from the blood facilitates the survival and rapid growth of malignant tumors, which need ample vascularization to enter the circulatory system [9]. Thus, it is important to note that the initiation of tumor angiogenesis is a crucial factor in tumor development [10]. Anti-angiogenic therapy (including gastric cancer, non-small-cell lung cancer, renal cell carcinoma, and colorectal cancer) has been approved for the treatment of multiple cancers [11–14]. However, studies have revealed that anti-angiogenic therapy provides only a short-term remission and tumor growth inhibition before drug resistance is developed [9]. Immunotherapy, such as immune checkpoint inhibitors (ICIs) for PD-1, PD-L1, and CTLA-4, is a promising treatment modality for diverse tumors, whose safety and effectiveness have been proven by a growing body of clinical studies [15, 16]. Accumulative evidence shows that the TME is responsible for tumor aggressive behaviors and influences immunotherapy efficacy [17]. The TME is mainly composed of tumor cells, blood vessels, infiltrating immune, and stromal cells [18]. The formation of neovascularization characterized by continuous and disordered is a characteristic of the TME. Interestingly, cross-talk between the tumor cells and angiogenesis mediates an immunosuppression microenvironment to promote immune escape of tumor cells, which seriously interferes with anti-tumor immunity and is an important reason for promoting tumor progression [19]. Hence, a comprehensive analysis of the association between angiogenesis and the TME can be used to recognize different tumor immunophenotypes and improve the predictive power of immunotherapy.

At present, several ARGs have been found to be involved in CRC development. The large proteoglycan versican (VCAN) is one of the main components of the extracellular matrix, which is involved in cell adhesion, proliferation, migration, and angiogenesis [20, 21]. Recent studies have demonstrated a positive correlation between VCAN and VEGF expression and microvessel density, with a worsened outcome in CRC patients with peritoneal metastasis [22]. Moreover, periosteum protein (POSTN), also known as osteoblast-specific factor 2, is a secreted extracellular matrix protein originally found in mesenchymal lineage cells (e.g., osteoblasts), and plays a key role in development and tissue regeneration [23]. There is a significant association between POSTN and the complete deletion of p53 in CRC tissues, and high POSTN expression is related to peritoneal and distant organ metastasis [24]. SERPINB5 (Serpin family B member 5) is a non-inhibitory member of the Serpin protease inhibitor superfamily with a variety of biological activities, including the regulation of cell adhesion, migration, apoptosis, oxidative stress response, and angiogenesis [25, 26]. SERPINB5 expression is significantly upregulated in CRC tissues, and is negatively correlated with progression-free survival of CRC patients [27, 28]. Dishearteningly, most current studies have focused on discovering the role of single ARG in CRC progression and prognosis. Moreover, the combination of the effects of multiple ARGs on TME infiltration characteristics of CRC have yet to be researched. Thus, a global analysis of the relationship between ARGs and TME, especially the tumor immune microenvironment, may provide the possibility of combining targeted therapy and immunotherapy to boost the predictive power of immunotherapy. On the other hand, reshaping the tumor immune microenvironment through a comprehensive understanding of the cross-talk between tumor angiogenesis and immune cells in the TME will contribute to the development of a strategy for a long-lasting anti-tumor immunity response.

Here, a comprehensive bioinformatic analysis of ARGs in CRC was performed by using TCGA and GEO databases. First, 1109 CRC patients were stratified into two discrete subtypes based on the ARGs expression. According to the differentially expressed genes (DEGs) identified in the two angiogenesis subtypes, we then identified three distinct gene subtypes and evaluated their molecular genetic features and prognostic value, as well as the abundance of infiltrating immune cells. We further established an ARG_score that precisely predicted the RFS of CRC patients and immunotherapeutic response. Finally, we observed evidence of a potential relationship between KLK10, *F.n*, and CRC based on whole genome microarray analysis. To further verify this association, we cultured two CRC cell lines *in vitro* with or without *F.n*. The results confirmed that the mRNA and protein levels of KLK10 were significantly upregulated by *F.n* infection at different time intervals. These findings not only extend the research community’s current understanding of ARGs in the CRC immunotherapy field but also provide a promising clinical biomarker and potential therapeutic target for *F.n*-infected CRC patients. We hope that our results will be helpful to the development of effective CRC immunotherapies.

## MATERIALS AND METHODS

### Data sources preparation and preprocessing

The microarray datasets that investigated the gene expression of CRC tissues were downloaded from the Gene Expression Omnibus (GEO) database, including GSE39582, GSE17536, and GSE38832. The mRNA expression profiles of 568 CRC tissues and 44 normal tissues were obtained from The Cancer Genome Atlas (TCGA) database. In addition, the relevant clinical data of the CRC patients were acquired using TCGA on November 8, 2021. The four datasets were preprocessed via the elimination of batch effects using the “ComBat” algorithm of the “SVA” package [29]. CRC patients with missing survival values were removed from further analysis. A total of 36 ARGs were gained from the MSigDB Team (Hallmark Gene set) (Supplementary Table 1).

### Unsupervised clustering analysis

Based on the expression of 36 ARGs, patients were identified as different molecular subtypes by k-means using the “ConsensusClusterPlus” package in R [30, 31]. In order to ensure the stability of the classification, the experiment was repeated 1000 times. Principal component analysis (PCA) was performed for the different clustering subtypes. The “GSVA” package in R was then used to carry out GSVA enrichment analysis [32].

### Association of molecular patterns with clinicopathologic factors and prognosis in CRC

To identification the clinical significance of the two subgroups produced by cluster analysis, we compared the relationships between molecular patterns and clinicopathological features. Furthermore, we evaluated the significance of RFS in distinct subtypes by using the “survival” and “survminer” packages in R [33].

### Evaluation of TME infiltration and immune checkpoints between different molecular patterns

The TME scores were assessed using the ESTIMATE algorithm, and TME cell infiltration was calculated using the CIBERSORT algorithm [34]. We also assessed the infiltrating immune cell scores and immune-related pathway activity by using the ssGSEA algorithm [35]. Furthermore, the association between the two subtypes on 47 immune checkpoints expression was analyzed [36–39].

### Identification of DEGs and functional enrichment analysis

The differentially expressed genes (DEGs) were identified by using the “limma” R package between the different angiogenesis subtypes (|log Foldchange (FC)| > 1 and false discovery rate (FDR) < 0.05). Further, the functions of these DEGs were performed by Gene Ontology (GO) analyses with the “clusterProfiler” package in R, which included biological process (BP), cellular component (CC), and molecular function (MF). Similarly, Kyoto Encyclopedia of Genes and Genomes (KEGG) analyses were also conducted to identify the significant enrichment pathways of these DEGs by the “clusterProfiler” package in R [40].

### Construction and validation of the angiogenesis-associated prognostic ARG_score

The ARG_score was constructed to evaluate the angiogenesis patterns of CRC patients. The DEGs expression data for different subtype groups (angiogenesis cluster A and angiogenesis cluster B) were standardized in CRC specimens and the intersect genes were selected. We first performed univariate Cox regression analysis to screen those DEGs associated with CRC RFS. Then, a deeper analysis was performed using unsupervised clustering method based on the expression of prognostic DEGs to classify patients into angiogenesis gene subtype A, angiogenesis gene subtype B, and angiogenesis gene subtype C. Subsequently, the CRC patients were randomly clarified into training (*n* = 555) and testing (*n* = 554) sets at a ratio of 1:1, and the former was selected to construct the angiogenesis-related prognostic ARG_score. In short, we performed the Lasso Cox regression algorithm to minimize the risk of overfitting by using the “glmnet” package in R based on prognostic genes associated with angiogenesis. We established a predictive model using LASSO Cox regression analysis: ARG_score = ∑Expi × βi, where Expi and βi represented each genes expression level and each genes coefficient index, respectively.

### RNA extraction, real-time PCR (RT-qPCR), and oligonucleotide transfection

The Trizol reagent (Invitrogen, Carlsbad, CA) was used to extract the total RNA from CRC cells (HCT116 and HT29) and tissues (six pairs CRC and nearby non-tumor tissues). Then, the PrimeScript RT reagent Kit (Takara, Japan) was performed to generate cDNA from 1 μg of total RNA. Finally, mRNA transcripts were quantified with a CFX-96 instrument RT-qPCR System (Bio-Rad Laboratories) and SYBR Green assays (Takara). SiRNA was purchased from GenePharma (Shanghai, China), and Lipofectamine 3000 (Invitrogen, USA) was used to perform transfection of siRNA. Moreover, nonspecific siRNA was used as negative controls, according to the manufacturer’s protocol. The 2^−ΔΔCT^ method was used to evaluate the relative target genes expression levels, normalizing with β-actin. The sequences of the primers used are shown in Supplementary Table 2.

### Clinical significance and stratification analysis of the prognostic ARG_score

The clinical characteristics of the CRC patients were isolated from the GEO and TCGA datasets. Then, a stratified analysis was performed to assess the predictive ability of the ARG_score in different subgroups. Further, univariate and multivariable Cox regression analyses were used to confirm the independent prognostic value of the ARG_score in training, testing, and entire sets. In addition, we examined the correlations between ARG_score and the 22 immune cells infiltrating levels, 47 immune checkpoints, tumor mutation burden (TMB), microsatellite instability (MSI), and cancer stem cells (CSC) index.

### Development of a nomogram for prediction

To explore independent prognostic value of the ARG_score, we established a nomogram prediction model for predicting the 1-year, 5-year, and 10-year RFS [41]. Moreover, the calibration maps described the precision of the nomogram in prognosis prediction (There are 1000 duplicate bootstrap methods).

### Mutation and drug sensitivity analysis

The TCGA-COAD/READ database was used to draw the 36 ARGs mutation frequency and oncoplot waterfall plot by using the “maftools” package in R [42]. Further, the location of CNV alteration of 36 ARGs on 23 chromosomes was produced by using the “Rcircos” package in R [43]. The semi-inhibitory concentration (IC50) values of chemotherapeutic drugs, which commonly used for CRC treatment were estimated by using the “pRRophetic” package in R [44]. Moreover, we used anti-angiogenic therapy and immunotherapy clinical trials (the gene expression profile and clinical manifestations of publicly available datasets) to explore the prediction ability of ARG_score for the therapeutic responses of tumor patients (GSE53127: a phase III study of metastatic CRC patients treated with bevacizumab [45], GSE103668: a phase II study of bevacizumab vs. platinum in patients with triple negative breast cancer [46], GSE78220: a phase III study of nivolumab vs. pembrolizumab in patients with previously treated metastatic melanoma [47], GSE126044: a phase II study of nivolumab vs. pembrolizumab in patients with non-small cell lung cancer [48], E-MTAB-3218 (ArrayExpress database, https://www.ebi.ac.uk/biostudies/arrayexpress/studies/E-MTAB-3218?accession=E-MTAB-3218#): a phase III study of nivolumab in patients with previously treated metastatic renal cell carcinoma [49]). The transcriptional information was adjusted and normalized by using the “edgeR” package in R [50], and the data were converted by using “voom” in the “limma” package in R [51]. Meanwhile, we also collated the prognostic and immunotherapy information of patients.

### Bacteria strains and cell lines

The *Fusobacterium nucleatum* strain ATCC 25586, purchased from American Type Culture Collection (ATCC, Manassas, VA, USA), was cultured overnight in brain heart infusion (BHI) broth under anaerobic conditions at 37°C. Human colorectal cancer cell lines HCT116 and HT29 were purchased from ATCC and cultured in McCoy’s 5A (Gibco, USA) supplemented with 10% fetal bovine serum (FBS) at 37°C in a humidified 5% CO2 atmosphere. Cells were seeded in 6-well plates and then incubated with or without bacteria at a multiplicity of infection (MOI) 100:1 [52, 53].

### Western blot analysis

Firstly, the cells were washed twice with ice-cold PBS and collected cell extracts with RIPA lysis buffer supplemented with Halt™ Protease Inhibitor Single-Use Cocktail (Thermo Fisher Scientific). Then, protein concentrations were quantified using BCA Protein Assay Kit (Thermo Fisher Scientific). Further, an equal amount of protein (10 μg) was separated by 10% SDS-PAGE and transferred to PVDF membranes (Invitrogen). The membranes were blocked in 1× TBS, 0.1% Tween-20 detergent (TBST) (Thermo Fisher Scientific) with 5% non-fat dry milk at room temperature for 2 h and then incubated with primary antibodies (E-cadherin (3195; Cell Signaling Technology), N-cadherin (13116; Cell Signaling Technology), Vimentin (5741; Cell Signaling Technology), β-actin (8457; Cell Signaling Technology)) overnight at 4°C. Next, after washing with TBST, the membranes were incubated with secondary antibodies for another 1 h at room temperature. Finally, the enhanced chemiluminescent (ECL) assay kit (Thermo Scientific, PA, USA) was applied for film visualization.

### Cell proliferation assay and wound healing assay

Firstly, approximately 1 × 10^4^ cells in 100 μl of medium were seeded in 96-well plates. Then, 10 μl of Cell Counting Kit-8 (CCK8, Yeasen, China) solution was added to each well at 24, 48, and 72 h. After an additional incubation period at 37°C for 3 h, the OD value was measured at 450 nm using a microplate reader. The wound healing assay was performed as previously described [54]. Briefly, the two cell lines (1 × 10^5^) were cultured in 6-well plates. The complete medium was replaced with a low concentration of serum fresh medium (2%) after 16 h. A 200-μl pipette tip was used to scratch across each well and wash gently with PBS two times. Meanwhile, multiple location markers were performed at the inoculated cells surface to compare the same wound area in future. The scratch area was photographed with an inverted microscope at 0, 24 and 48 h, respectively. The experiment was conducted in triplicate.

### Statistical analysis

R (v4.3.1), GraphPad Prism (version 10.0), and SPSS (27.0) were used to perform all statistical analyses. A *p*-value less than 0.05 was considered statistically significant.

### Availability of data and materials

In this study, publicly available datasets were used to perform analyses, as described in the materials and methods. The datasets are available from TCGA (https://portal.gdc.cancer.gov/) and Gene Expression Omnibus (https://www.ncbi.nlm.nih.gov/geo/), including GSE39582, GSE17536, and GSE38832.

## RESULTS

### Overview of the genetic and transcriptional alterations of ARGs in CRC

**Figure 1:**
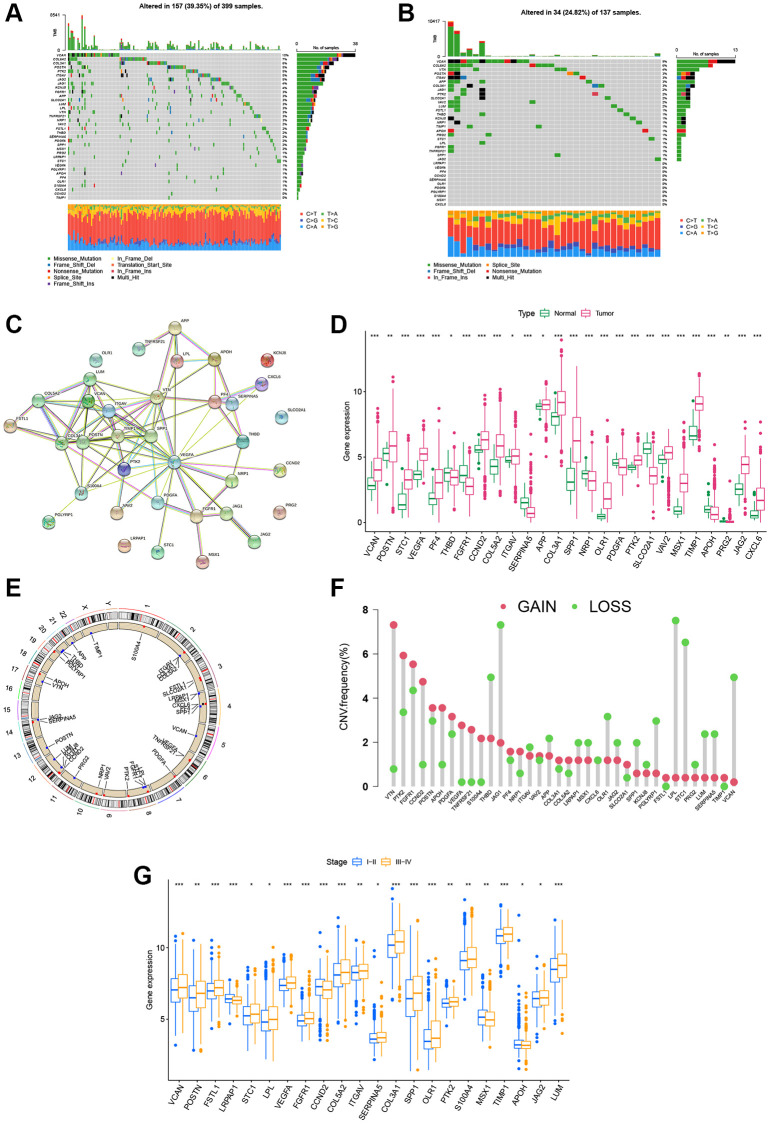
**Landscape of the ARG genetic alterations in CRC.** (**A**, **B**) Mutation frequencies of 36 ARGs in 399 and 137 patients with COAD and READ based on the TCGA cohort, respectively. (**C**) PPI network showing the interactions of ARGs (the minimum required interaction score was 0.4). (**D**) Expression distributions of DEGs between tumor and normal tissues. (**E**, **F**) Locations of CNV alterations in ARGs on 23 chromosomes and the frequencies of CNV gain, loss, and non-CNV among ARGs, respectively. (**G**) Expression distributions of DEGs between the high- and low-stage groups. ^*^P < 0.05; ^**^P < 0.01; ^***^P < 0.001.

Firstly, the somatic mutations pattern of 36 ARGs in CRC cohort (COAD and READ samples) was investigated. The Figure 1A demonstrated that 157 (39.35%) had mutations of these ARGs in 399 COAD cohort. Notably, VCAN was the gene with the highest mutation frequency (10%), however, there were no mutations in two ARGs (CCND2 and TIMP1). In addition, the Figure 1B showed that 34 (24.82%) had mutations of these ARGs in 137 READ cohort, and VCAN was also the gene with the highest mutation rate (9%). Further, the protein–protein interaction (PPI) analysis was performed to construct, and indicated that VEGFA, SPP1, POSTN, VTN, COL3A1, ITGAV, and TIMP1 were the hub genes (Figure 1C). We then performed to compare the mRNA expression levels between normal and tumor tissues. A total of 26 DEGs were determined, most of which were rich in the tumor tissues (Figure 1D). Moreover, the somatic copy number alterations of ARGs were investigated, and the copy number variation (CNV) alterations on their respective chromosomes were demonstrated in Figure 1E. As shown in Figure 1F, 36 ARGs demonstrated evident CNV alterations. Notably, there was a positive association between the levels of ARGs and CNV alteration; for example, the expression levels of PRG2, SERPINA5, and THBD were low in tumor tissues, while VEGFA, PF4, and PTK2 were expressed at high levels. These results indicate that CNV may modulate the expression of ARGs in CRC. According to clinical characteristics, the CRC patients were divided into early-stage groups (I + II) and advanced-stage groups (III + IV). As shown in Figure 1G, there were significant differences between the two groups in 36 ARGs. The expression levels of 12 ARGs (VEGFA, VCAN, POSTN, STC1, COL5A2, ITGAV, COL3A1, SPP1, OLR1, PTK2, TIMP1, and JAG2) were higher in both CRC tissues and the advanced-stage groups, which indicated that their potential function as carcinogenic genes in CRC.

### Identification of angiogenesis patterns in CRC

**Figure 2:**
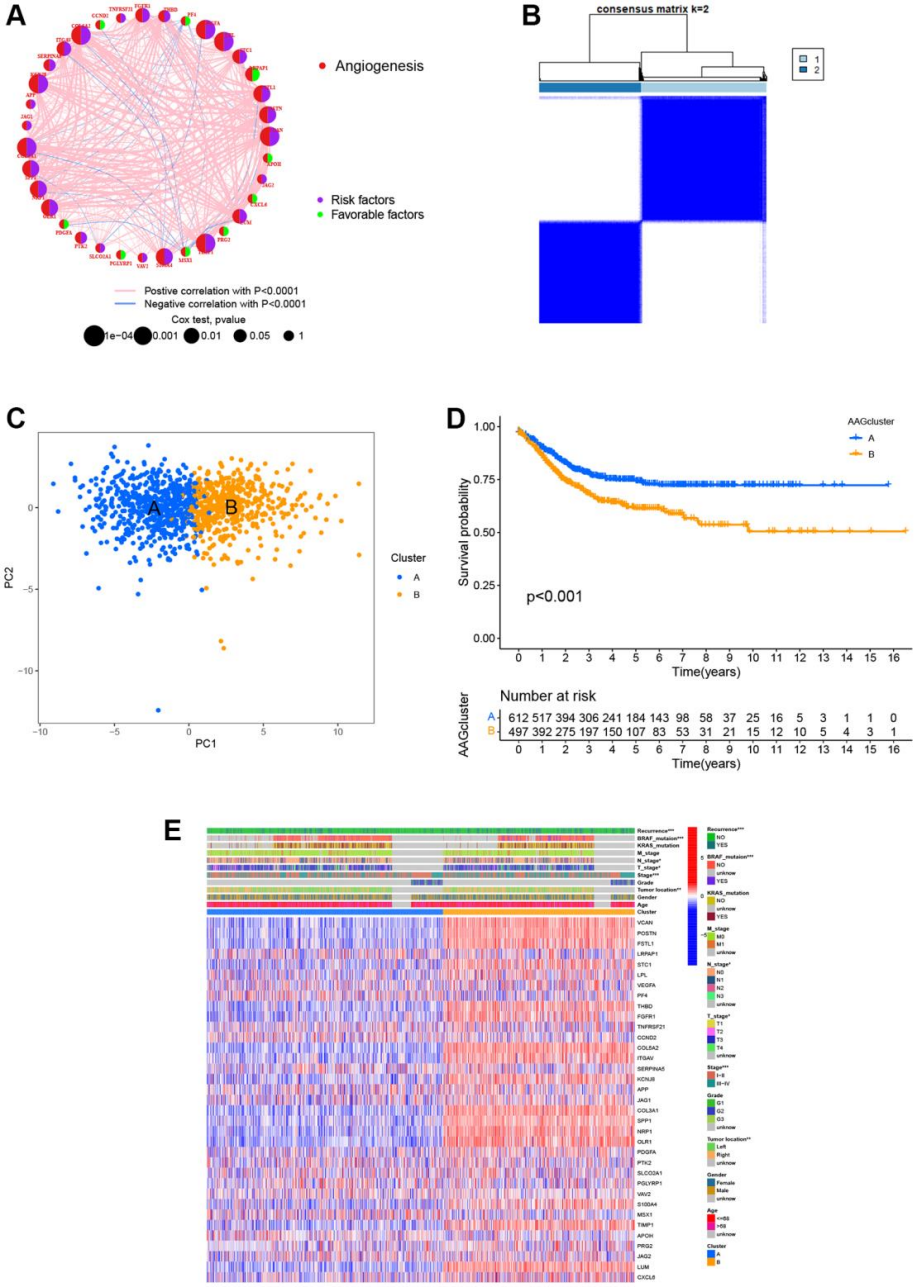
**ARG-related subtypes and clinicopathological and biological characteristics of two distinct subgroups of samples divided by consistent clustering.** (**A**) The correlation network of ARGs in CRC (red line: positive correlation; blue line: negative correlation). (**B**) Consensus matrix heatmap defining two clusters (k = 2) and their correlation area. (**C**) PCA showing a remarkable difference in transcriptomes between the two subtypes. (**D**) Univariate analysis showing 36 ARGs related to the RFS time. (**E**) Differences in clinicopathological and biological characteristics of the two distinct subtypes. ^*^P < 0.05; ^**^P < 0.01; ^***^P < 0.001.

Supplementary Figure 1 displays the article framework and workflow. Our study integrated 1109 patients from four eligible CRC datasets to identify potential angiogenesis patterns of CRC. Detailed clinical information of 1109 patients are presented in Supplementary Table 3. We extracted the expression levels of 36 ARGs and then revealed their prognostic value using uniCox and Kaplan–Meier analysis [55] (Supplementary Table 4). Next, we established the angiogenesis network to visualize the landscape of 36 ARGs, including their interactions, connections, and prognostic values (Figure 2A, Supplementary Table 5). Based on these ARGs, we identified two different angiogenesis-related patterns by consensus clustering, including 612 cases in Cluster A and 497 cases in Cluster B (Figure 2B). Further, PCA analysis confirmed that the CRC patients were well-differentiated when k = 2, indicating the robust and reliable clustering of the samples (Figure 2C). The Kaplan–Meier curves demonstrated that the survival outcome of Cluster A was better than that of Cluster B (*P* < 0.001, Figure 2D). Furthermore, the heatmap was used to visualize the relationship between 36 ARGs expression and clinical features. Compared to Cluster B, we found that Cluster A was markedly associated with early pathological stage (*P* < 0.001), left-sided CRC (*P* < 0.01), without BRAF mutations (*P* < 0.001), and low risk of recurrence (*P* < 0.001, Figure 2E).

### Variation in characteristics of TME infiltration between two angiogenesis subtypes

**Figure 3:**
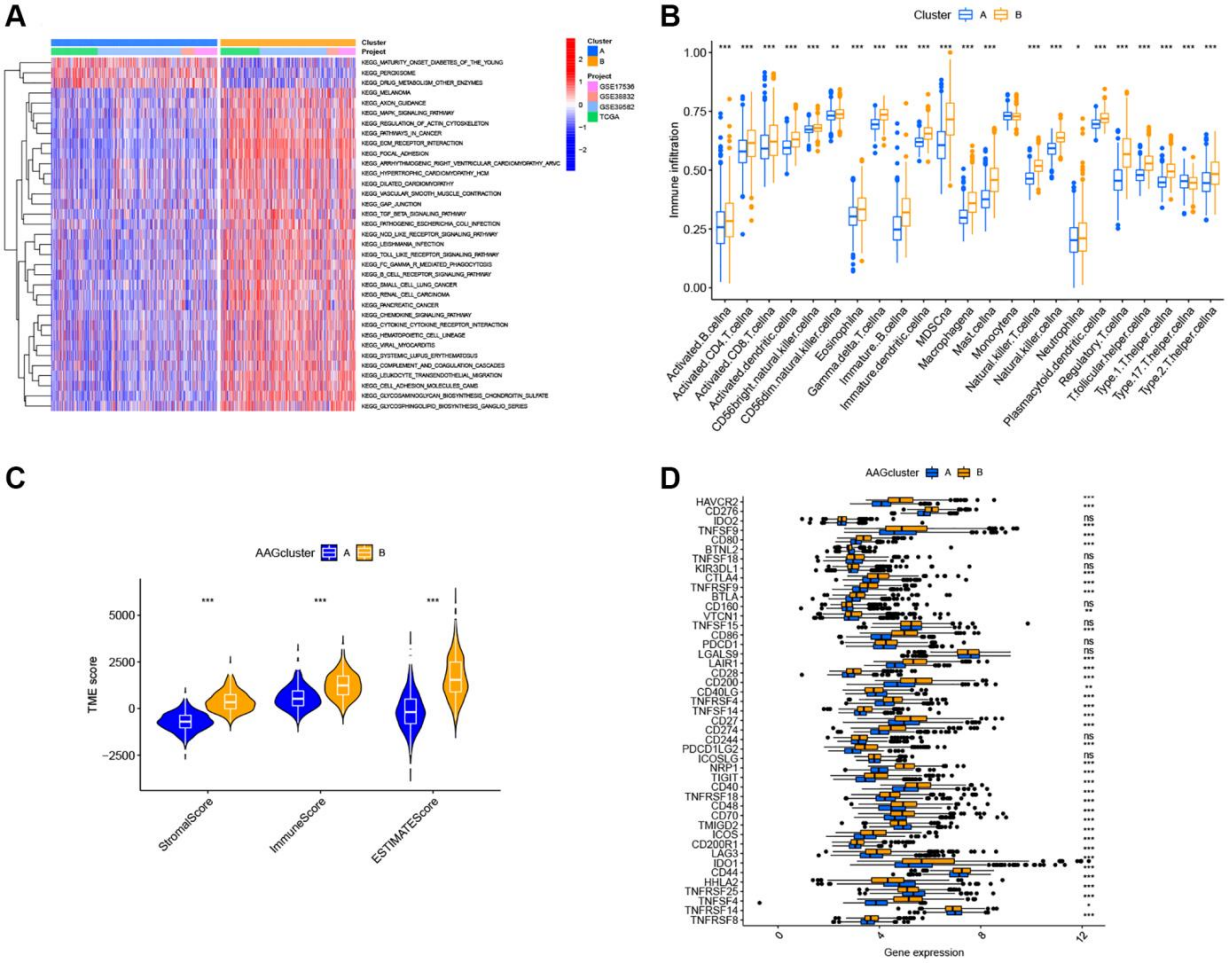
**Two ARG subtypes showed diverse tumor immune cell microenvironments.** (**A**) Biological processes analyzed by GSVA showing the active biological pathways in distinct subtypes. (**B**) The abundance of 23 TME infiltrating cells between the two subtypes of ARGs. (**C**) Correlations between the two ARG subgroups and the TME score. (**D**) Expression levels of 47 immune checkpoints in the two subtypes. ^*^P < 0.05; ^**^P < 0.01; ^***^P < 0.001.

GSVA enrichment analysis showed that cluster B was significantly enriched in cancer-associated pathways (e.g., renal cell carcinoma, glioma, and small cell lung cancer), metastasis-associated pathways (e.g., cell adhesion molecules cams, ECM receptor interaction, and focal adhesion), and immune fully-activated pathways (e.g., B cell receptor signaling pathway, cytokine receptor interaction, chemokine signaling pathway activation, and the NOD-like and Toll-like receptor signaling pathways) (Figure 3A). To further investigate the correlation between ARGs and the TME of CRC, we analyzed the infiltrating levels of 23 human immune cell subsets in distinct subtypes with the CIBERSORT algorithm. We observed that, except for the monocyte cells, the other 22 types of immune cells were all poorly activated in Cluster A (Figure 3B). Further, we analyzed the differences in terms of the immune score, stromal score, and estimate score between Cluster A and Cluster B using the ESTIMATE algorithm. Our results revealed that Cluster B had higher TME scores than Cluster A, which indicates that Cluster B had a higher levels of angiogenesis-dependent extracellular matrix components (*P* < 0.001, Figure 3C). Moreover, we analyzed the expression of 47 important immune-oncology targets between Cluster A and Cluster B. As shown in Figure 3D, significant differences were observed in 36 immune checkpoints between the two subtypes, including PD-L1, CTLA-4, and LAG3 (*P* < 0.001). Altogether, our findings suggest that ARGs were involved in the formation of TME infiltration and represent different prognostic features in patients of CRC.

### Generation of gene subtypes based on two angiogenesis clusters

**Figure 4:**
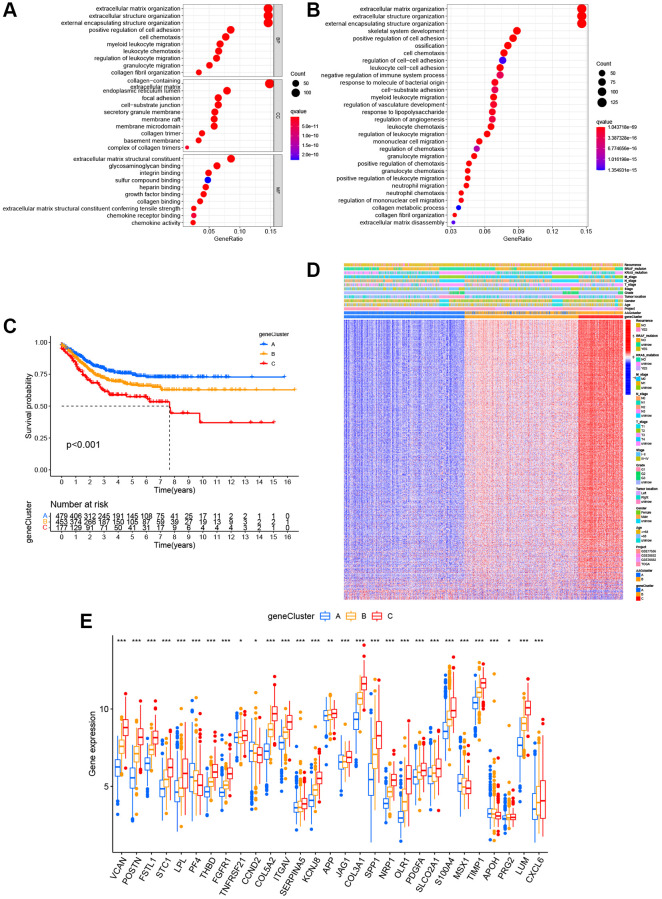
**Identification of gene subgroups based on DEGs.** (**A**, **B**) The bubble graph for Gene Ontology (GO) analysis and the barplot graph for Kyoto Encyclopedia of Genes and Genomes (KEGG) analysis in the two ARG subtypes. (**C**) Kaplan–Meier curves for the RFS of the three gene subtypes. (**D**) Relationships between clinicopathologic characteristics and the three gene subtypes. (**E**) Differences in the expression of 36 ARGs among the three gene subtypes. ^*^P < 0.05; ^**^P < 0.01; ^***^P < 0.001.

To confirm the biological behavioral differences between these two angiogenesis subtypes, 927 angiogenesis clusters related DEGs were obtained using the “limma” package in R to obtain insights into their biological function. These angiogenesis subtypes related DEGs were mainly enriched in biological metastasis processes (Figure 4A). KEGG analysis showed enrichment of immune-, cancer- and metastasis-related pathways (Figure 4B), indicating that angiogenesis plays a vital role in the immune regulation of the TME and the modulation of tumor metastasis. Next, uniCox analysis was performed to explore the prognostic value of these DEGs and 620 genes related to RFS time were extracted (*P* < 0.05, Supplementary Table 6). To further understand specific regulation mechanism, a consensus clustering method was performed to divide patients into different gene clusters (named gene_Cluster A, B, and C, respectively) based on prognostic genes (Supplementary Figure 2). Kaplan-Meier curves indicated that patients in gene_Cluster C showed the worst RFS, whereas patients in gene_Cluster A had a favorable RFS (*P* < 0.001, Figure 4C). Furthermore, angiogenesis related gene_Cluster C was related to the advanced TNM stage, KRAS and BRAF mutations, and a higher recurrence risk (Figure 4D). Furthermore, the angiogenesis-related gene clusters demonstrated significant differences in ARG expression, which were consistent with the expected results of the angiogenesis clusters (Figure 4E).

### Construction and validation of the prognostic ARG_score in CRC

**Figure 5:**
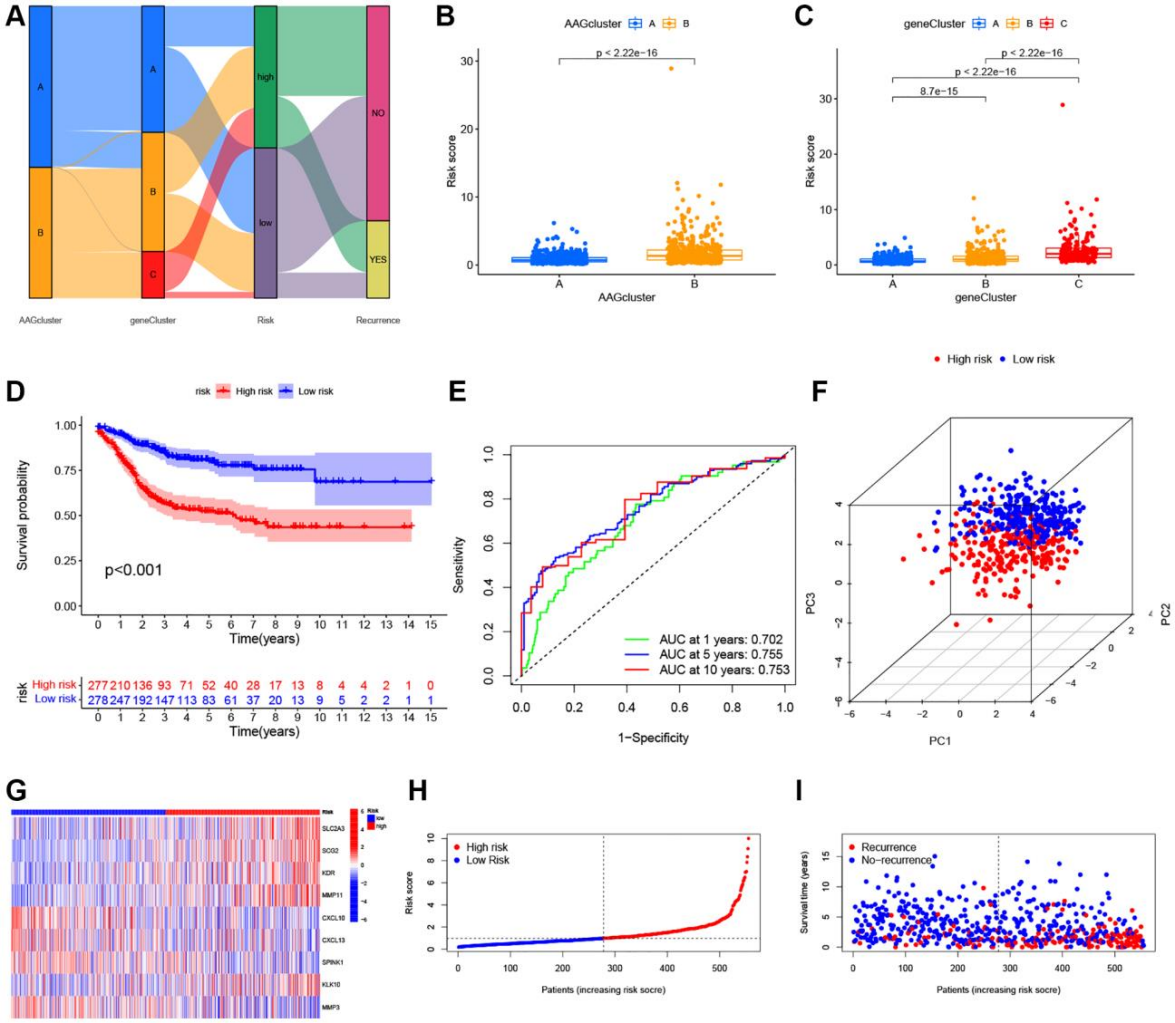
**Construction of the ARG_score in the training set.** (**A**) Alluvial diagram of subtype distributions in groups with a different ARG_score and prognosis. (**B**) Differences in ARG_score between the two ARGs clusters. (**C**) Differences in ARG_score among the three gene subtypes. (**D**) Kaplan–Meier analysis of the RFS between the low- and high-ARG_score groups. (**E**) ROC curves to predict the sensitivity and specificity of 1-, 5-, and 10-year survival according to the ARG_score. (**F**) PCA analysis based on the prognostic model. (**G**–**I**) The distribution of the risk score, survival time, and survival status, and the heatmap of the 9 selected prognostic genes between the high- and low-ARG_score groups, respectively.

The ARG_score was constructed based on the DEGs associated with angiogenesis clusters. First, we randomly assigned the patients into training (*n* = 555) and testing (*n* = 554) groups at a ratio of 1:1. LASSO and multivariate Cox analyses for 620 angiogenesis cluster-related prognostic DEGs were then used to identify an optimal prognostic signature (Supplementary Figure 3). A total of 9 RFS-associated genes were identified based on the minimum partial likelihood deviance, and the ARG_score for each patient in the training, testing, and entire datasets were calculated based on the risk formula, as follows: risk score = (expression of SLC2A3 × 0.2758) + (expression of SCG2 × 0.1617) + (expression of KDR × 0.3094) + (expression of MMP11 × 0.1725) + (expression of CXCL10 × −0.2112) + (expression of CXCL13 × −0.0965) + (expression of SPINK1 × −0.1023) + (expression of KLK10 × 0.0877) + (expression of MMP3 × −0.1427). A Sankey diagram was constructed to visualize the patients’ distribution in the two angiogenesis clusters, three gene clusters, and two ARG_score groups (Figure 5A). In addition, significant differences were observed in the ARG_score of the angiogenesis clusters and gene clusters (Figure 5B, 5C). The highest and lowest ARG_score was observed in gene_Cluster C and gene_Cluster A, respectively, suggesting a low ARG_score may be closely associated with immune activation-associated features. According to the results of survival analysis, we found that the higher ARG_score of both classifications were associated with a worse RFS. Moreover, Kaplan-Meier analysis implied that low ARG_score patients had a better RFS over patients with a high ARG_score in the training cohort (*P* < 0.001, Figure 5D), and the AUCs of the 1-, 5-, and 10-year RFS were 0.702, 0.755, and 0.753 in the training cohort, respectively (Figure 5E). PCA analysis demonstrated a clear distribution between the low- and high-ARG_score groups (Figure 5F). The relationships between the 9 selected prognostic signatures and the ARG_score can be witnessed in the heatmap (Figure 5G). Meanwhile, the distribution of the ARG_score and RFS time of patients is shown in Figure 5H, 5I. To assess the predictive robustness of the risk model, the ARG_score of the testing cohort and the entire cohort were obtained (Supplementary Figures 4, 5). Based on the median score of the training cohort, we also separated patients into different risk subgroups. Also, survival analysis revealed a superior RFS of low score patients compared to high score patients (*P* < 0.001). Further, the classification efficiency analysis of 1-, 5- and 10-years survival prediction showed that ARG_score had a high AUC values, suggesting that the model had the ability to evaluate the survival of CRC patients.

### Validation of the 9 prognostic ARGs in CRC tissues

To more effectively verify our findings, the expression levels of 9 prognostic signatures were measured in 6 pairs of tumor tissues and normal adjacent tissue samples by RT-qPCR. As shown in Supplementary Figure 6I–6O, the expression levels of SLC2A3, MMP11, CXCL10, KLK10, and MMP3 were upregulated, while those of SCG2 and CXCL13 were downregulated in CRC tissues compared to normal tissues. No statistically significant difference was observed in the mRNA levels of KDR and SPINK1 between the normal and tumor tissues (data not shown). These experimental results were consistent with those predicted by the bioinformatics methods (Supplementary Figure 6A) and the GEPIA database (Supplementary Figure 6B–6H).

### Stratified analysis and independent prognostic value of ARG_score

To determine the influence of the ARG_score on clinical features, the association between ARG_score and clinical factors was investigated in CRC patients, including age, gender, TNM stage, tumor location, KRAS, and BRAF mutation status. We found a higher ARG_score in patients in the stage III–IV subgroup than those in the stage I–II subgroup (*P* < 0.001, Supplementary Figure 7A). To determine whether the ARG_score could be used as an independent prognostic factor, we carried out univariate and multivariate Cox regression analyses by using ARG_score and multiple clinical characteristics. As shown in Supplementary Figure 7B–7D, ARG_score was an independent predictor of CRC outcome in the training cohort, with consistent results observed across the testing cohort and the entire cohort (*P* < 0.001). To further investigate the prognostic significance of the ARG_score in CRC, we performed a stratified analysis in different clinical parameter subgroups. As shown in Supplementary Figure 8, CRC patients in the high ARG_score group tended to have a shorter RFS than that in the low ARG_score group (*P* < 0.001), as demonstrated by the results for age, sex, tumor location, TNM stage, and KRAS and BRAF mutations.

### Establishment of a clinical nomogram for the prediction model in CRC

**Figure 6:**
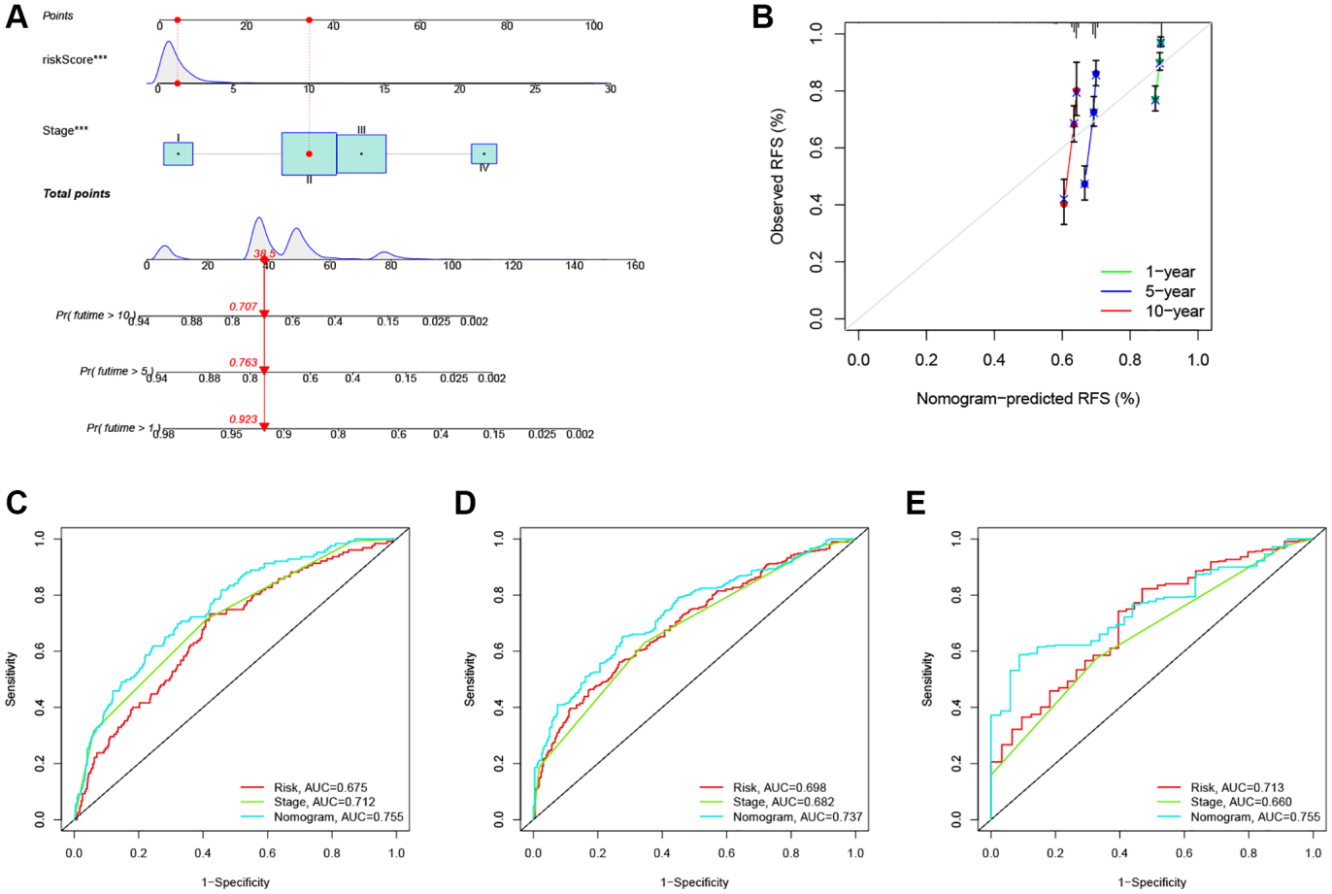
**Construction and evaluation of a predictive nomogram.** (**A**) The nomogram predicts the probability of the 1-, 5-, and 10-year RFS. (**B**) The calibration plot of the nomogram predicts the probability of the 1-, 3-, and 5-year RFS. (**C**–**E**) The time-dependent ROC curves of the nomograms compared for the 1-, 5-, and 10-year RFS in CRC, respectively. ^***^P < 0.001.

Given the high correlation between the ARG_score and patients’ prognosis in CRC, we further performed to establish a nomogram by using multivariate Cox regression to predict the 1-, 5-, and 10-year RFS (Figure 6A). Calibration plots showed that 1 -, 5 -, and 10-year RFS could be predicted relatively well (Figure 6B). Further, the AUC values of the nomogram with that of the TNM stage were estimated for predicting the 1-, 5-, and 10-year RFS, respectively. Compared with TMN staging features, the prediction ability of ARG_score in the nomogram was superior (Figure 6C–6E).

### Relationship between ARG_score and TME infiltration in CRC

**Figure 7:**
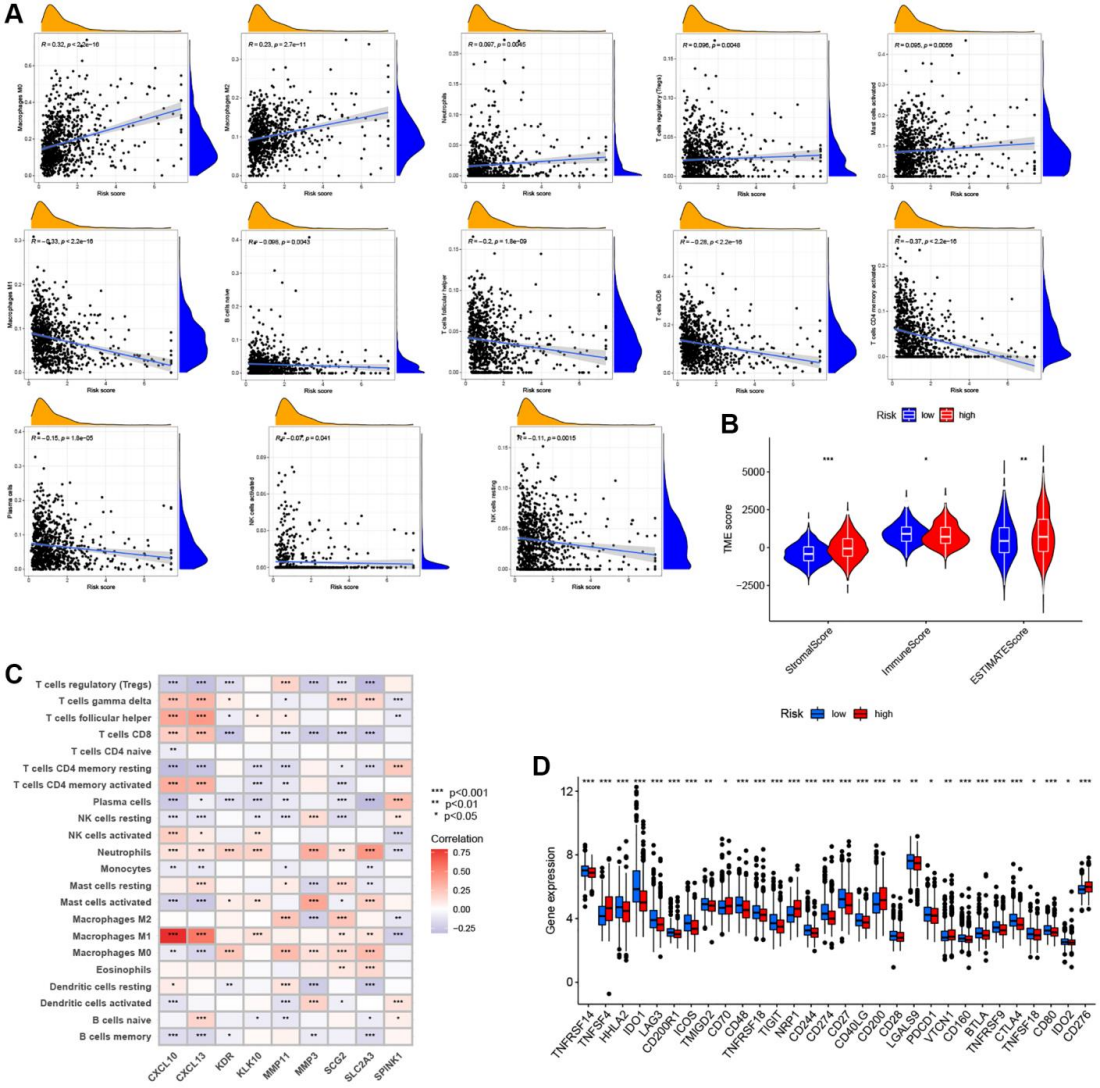
**Evaluation of the TME and immune checkpoints between the distinct ARG_score groups.** (**A**) Correlations between ARG_score and immune cell types. (**B**) Correlations between ARG_score and both immune and stromal scores. (**C**) Correlations between the abundance of immune cells and 9 selected genes in the proposed model. (**D**) Expression of 47 immune checkpoints in the low- and high-ARG_score groups. ^*^P < 0.05; ^**^P < 0.01; ^***^P < 0.001.

There is increasing evidence that TME cell infiltration is critical to tumor development and therapeutic response. Thus, we used the CIBERSORT algorithm to investigate the abundance of different immune cell types between the low- and high-ARG_score groups. As shown in Figure 7A, the ARG_score was positively correlated with the infiltration of M0 and M2 macrophages, neutrophils, regulatory T cells (Tregs), and activated mast cells, while being negatively correlated with the infiltration of M1 macrophages, naïve B cells, follicular helper T cells, CD8 + T cells, CD4 + memory-activated T cells, plasma cells, activated NK cells, and resting NK cells. In addition, a low ARG_score was also closely related to a high immune score, whereas a high ARG_score was linked to a high stromal score (Figure 7B). We then evaluated the potential relationship between the 9 selected genes in the prognostic signature and the abundance of immune cells, and found that most immune cells were significantly associated with these 9 genes (Figure 7C). Furthermore, we evaluated the correlations between 47 immune checkpoints and this prognostic signature. As shown in Figure 7D, 30 immune checkpoints were discrepantly represented in the two groups, including PD-L1, CTLA-4, and LAG3.

### The ARG_score had observable correlations with the TMB, MSI, and CSC indices

**Figure 8:**
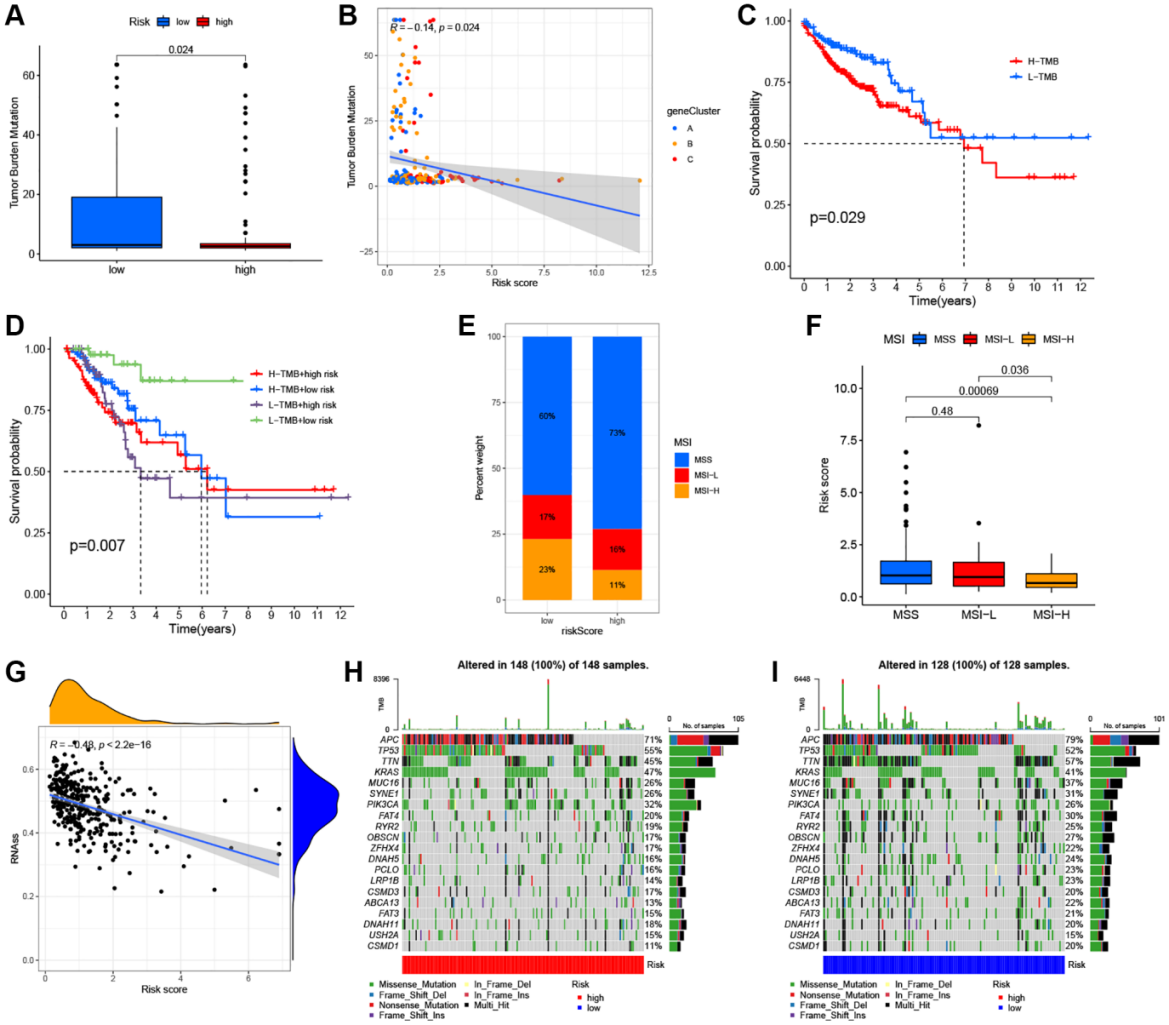
**Comprehensive analysis of the ARG_score in CRC.** (**A**, **B**) Relationships between the ARG_score and TMB. (**C**) Kaplan–Meier analysis of the RFS between the low- and high-TMB groups. (**D**) Survival analysis among four groups stratified by both TMB and ARG_score in CRC. (**E**, **F**) Relationships between ARG_score and MSI. (**G**) The correlation of the ARG_score with CSC index. (**H**, **I**) The waterfall plot of somatic mutation features established with the high- and low-ARG_score groups.

Several studies have reported that patients with high TMB or MSI-H status may be more sensitive to immunotherapy [5, 56, 57]. As shown in Figure 8A, a higher TMB was observed in the low ARG_score groups compared to the high ARG_score groups (*P* < 0.05), indicating that low ARG_score patients may benefit more from immunotherapy drugs. In addition, our findings indicate that the ARG_score was negatively correlated with TMB based on Spearman correlation analysis (R = −0.14, *P* < 0.05, Figure 8B). We further evaluated the potential relationship between TMB status and RFS, and found significant differences in clinical outcome between patients with high and low TMB groups (*P* < 0.05, Figure 8C). Meanwhile, this prognostic benefit in the high TMB groups was more affected by the ARG_score than that of the low TMB groups (*P* < 0.01, Figure 8D). Furthermore, correlation analysis showed that the high ARG_score group was associated with a microsatellite stable (MSS) state, while the low ARG_score group was associated with a high microsatellite unstable (MSI-H) state (Figure 8E, 8F). This result also implies that the low ARG_score groups benefitted from immunotherapy, with consistent results observed in the TMB analyses. Furthermore, the relationships between ARG_score and cancer stem cell (CSC) index was analyzed. As shown in Figure 8G, the results showed that ARG_score was negatively correlated with CSC index values (R = −0.48, *P* < 0.001), meaning that a lower ARG_score was associated with more pronounced stem cell characteristics and lower degree of cell differentiations. Additionally, the distribution differences of the top 20 somatic mutations genes among distinct ARG_score groups were analyzed based on TCGA-COAD cohort. As shown in Figure 8H, the mutation incidences of APC, TP53, TTN, KRAS, MUC16, SYNE1, PIK3CA, and FAT4 were higher than or equal to 20% in the high ARG_score groups. Surprisingly, except for the USH2A, all other mutated genes were higher than or equal to 20% in the low ARG_score groups (Figure 8I). Taken together, our results indicate that these genes were more likely to be mutated in the low ARG_score groups compared to the high ARG_score groups.

### Screening of the sensitive molecular drugs and prediction of the immunotherapy efficacy

**Figure 9:**
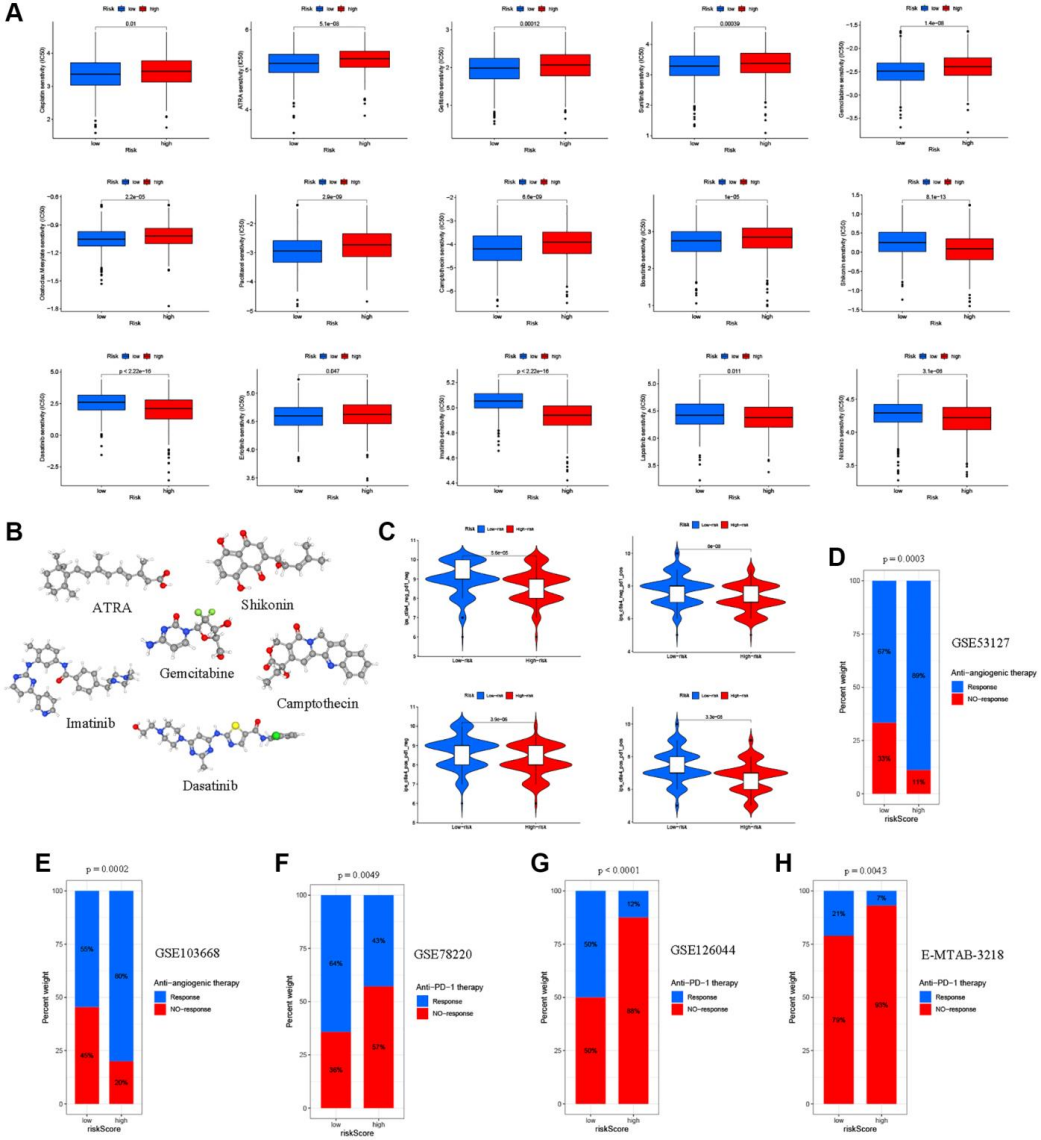
**Relationships between the ARG_score and therapeutic sensitivity.** (**A**) Relationships between the ARG_score and chemotherapy and sensitivity to targeted inhibitor therapy. (**B**) The 3D structure of 6 potential target drugs screened out from the cMap database. (**C**) The IPS in the different ARG_score groups. (**D**, **E**) Proportion of patients with different treatment outcomes (bevacizumab therapy) in the low- and high-ARG_score groups. The proportion of response patients in the low ARG_score groups were significantly higher than that in the high-ARG_score groups in both the GSE53127 and GSE103668 cohorts. (**F**–**H**) Patients with a high ARG_score exhibited poorer response outcomes after immunotherapy (anti-PD-1 therapy) using the GSE78220, GSE126044, and E-MTAB-3218 cohorts.

Currently, CRC patients usually receive chemotherapy, targeted therapy, and immunotherapy after radical surgery, which has been shown to significantly improve clinical outcomes [58]. To explore the potential therapeutic drugs of patients in the low- and high-ARG_score groups, we analyzed the IC50 values of common therapeutic drugs in CRC patients. Our data showed that the low ARG_score groups may positively react to cisplatin, ATRA, gefitinib, sunitinib, gemcitabine, obatoclax, mesylate, paclitaxel, camptothecin, and bosutinib, while the high ARG_score groups may respond better to shikonin, 11asatinib, erlotinib, imatinib, lapatinib, and nilotinib (Figure 9A). The 15 associated candidate drugs were ranked by the *p*-value of differences, and top 6 most associated molecule drugs were screened for CRC patients. The 3D structures of ATRA, gemcitabine, camptothecin, shikonin, imatinib, and 11asatinib were displayed through the PubChem database (Figure 9B). The vascular endothelial growth factor (VEGF) pathway has been shown to play a critical role in the control of CRC angiogenesis [59]. Anti-angiogenic drugs targeting the VEGF signaling pathways, including bevacizumab, has been shown to be an effective and tolerable therapy that improves survival in advanced CRC patients [60]. We further investigated the ability of the ARG_score to predict response to bevacizumab therapy in the GSE53127 cohort. As shown in Figure 9D, the high ARG_score groups had a higher objective response rate compared to the low ARG_score groups (*P* < 0.001). Also, in the GSE103668 cohort, the proportion of response patients in the high ARG_score groups were significantly higher than that in the low ARG_score groups (*P* < 0.001, Figure 9E). Furthermore, we observed significant negative associations between immune checkpoint levels (e.g., PD-1/L1, CTLA-4, and LAG3) and the ARG_score during the evaluation of ARG-related TME characteristics. Thus, we used two methods to assess the predictive ability of the ARG_score in the immunotherapy response. The immunophenoscore (IPS) has been applied to predict the immunotherapeutic benefits to CTLA-4 and PD-1. Our results indicated that the low ARG_score group had a good response to PD-1 and CTLA-4 single positive, double positive, and double negative (Figure 9C). Further, three datasets (GSE78220, GSE126044, and E-MTAB-3218 cohorts) containing patient pretreatment and immunotherapeutic (anti-PD-1 therapy) data were collected, which had various clinical (PR/CR: responses or PD/CD: no-responses) and transcriptional (RNA-seq) information. As shown in Figure 9F–9H, the proportion of response patients in the low ARG_score groups were significantly higher than that in the high ARG_score groups (*P* < 0.05), which is consistent with the characteristics of significant vascular infiltration and immunosuppression in the high ARG_score groups. Together, our findings suggest that the ARG_score model had greater potential for predicting immunotherapy efficacy.

### KLK10 was identified as a prognostic key ARG and immunotherapy target

**Figure 10:**
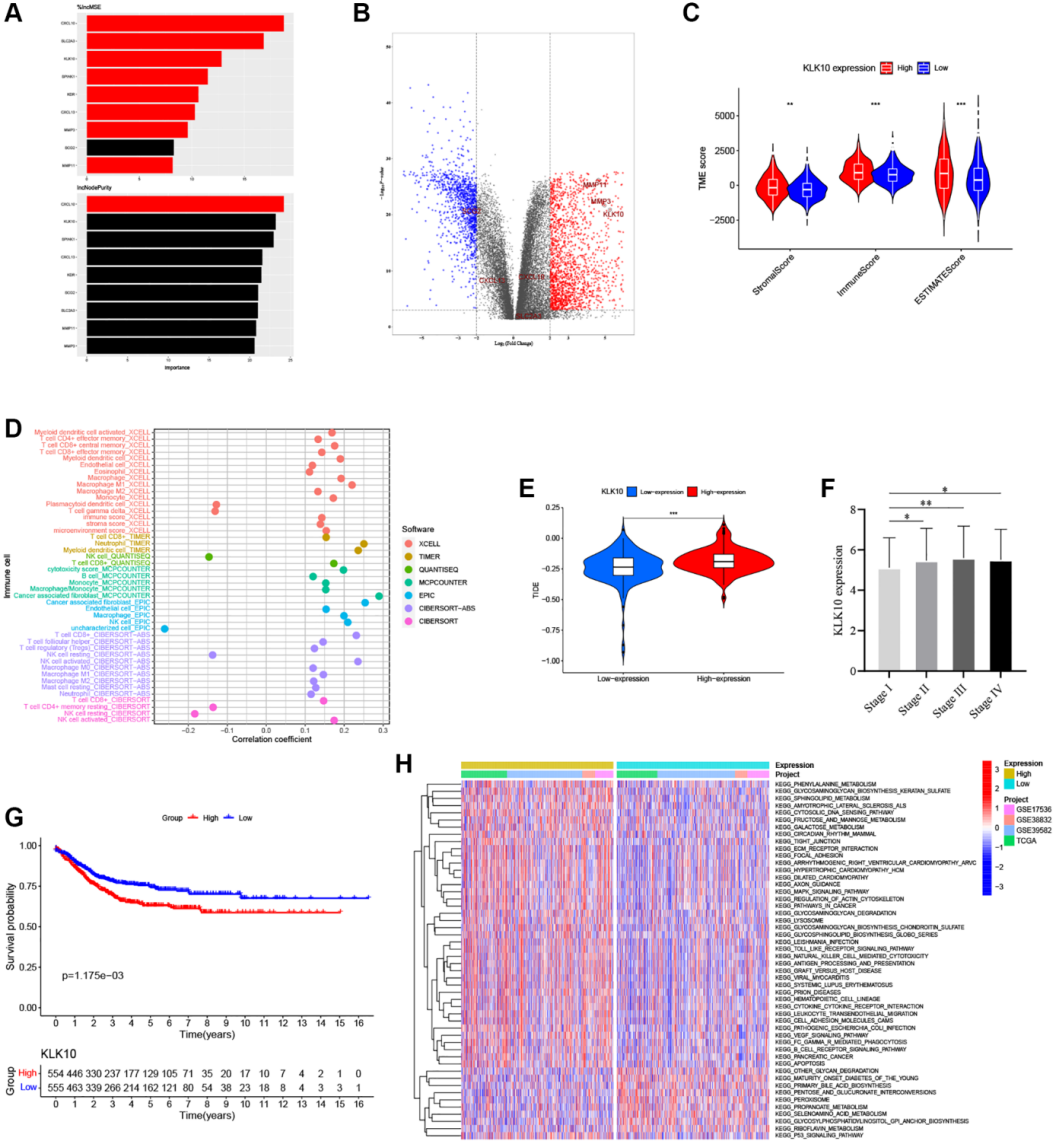
**Identification of the key biomarkers in ARGs based on multi-methods.** (**A**) The variable importance of 9 prognostic ARGs in RF models. (**B**) Volcano plot of significant DEGs between the normal and tumor tissues in four CRC cohorts (red represents upregulated genes, blue indicates downregulated genes, and gray represents no change). (**C**) Correlations between KLK10 expression and both the immune and stromal scores. (**D**) Spearman correlation analysis performed using different algorithms between KLK10 expression and immune cell infiltration. (**E**) The relationship between KLK10 expression and the TIDE score. (**F**) The correlation of KLK10 expression with clinicopathological staging characteristics. (**G**) Survival analysis for CRC patients with distinct KLK10 expression. (**H**) The GSVA pathway enrichment analysis between distinct KLK10 expression groups. ^*^P < 0.05; ^**^P < 0.01; ^***^P < 0.001.

According to the expression of 9 prognostic ARGs, a machine learning algorithm was used to predict candidate ARGs based on survival and death as binary dependent variables. As shown in Figure 10A, the three most important ARGs were CXCL10, SPINK1, and KLK10 in the RF model. Further, we analyzed the expression levels of these ARGs by combining the GTEx and TCGA databases. Our results showed that KLK10 was the most significantly different gene between normal and tumor tissues (Figure 10B, FDR < 0.001 and log_2_ FC > 5), which showed a high degree of consistency with the results of qRT-PCR analysis (Supplementary Figure 6N). To investigate the relationship between KLK10 expression and TME infiltration, we assessed the stromal score and immune score based on ESTIMATE analysis. As shown in Figure 10C, the stromal and immune score of high KLK10 expression groups were higher than those of low KLK10 expression groups, indicating that KLK10 expression was related to a hot TME. Additionally, we performed different algorithms to determine the relationship between KLK10 expression and infiltration levels of different immune cell types. The lollipop shape demonstrated that most immune cells had a positive correlation with KLK10 expression (Figure 10D). Furthermore, we used the TIDE algorithm to dissect the relationship between KLK10 expression and immunotherapy response. The results indicated that high KLK10 expression was positively correlated with the tumor immune escape (T cell dysfunction and T cell exclusion), which provides novel insights into precision immunotherapy in CRC (Figure 10E). Further, we explored the relationship between clinical and prognostic value and KLK10 expression, and found that the expression of KLK10 was significantly related to the pathologic stage (Figure 10F). Moreover, high KLK10 expression was markedly related to poor RFS based on the median of the KLK10 expression levels (Figure 10G), highlighting its potential function as the tumor promotor in the occurrence and development of CRC.

### Exploration and validation of the biological functions of KLK10 in CRC

**Figure 11:**
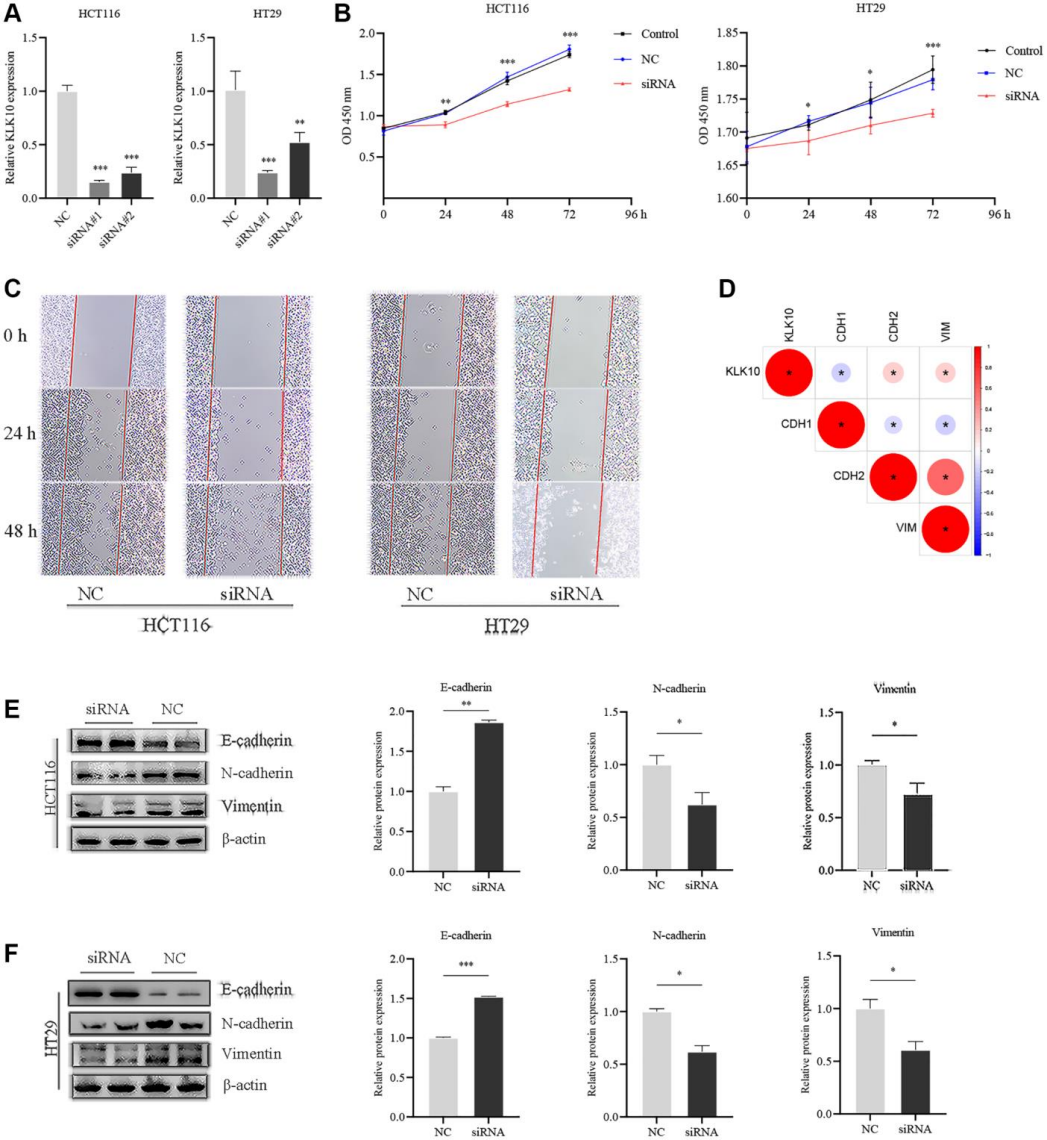
**Influences of KLK10 expression on CRC cell proliferation, migration, and invasion.** (**A**) The efficacy of the KLK10 transcript was detected after KLK10 knockdown in two CRC cell lines. (**B**) Cell viability was assessed by CCK8 array when KLK10 expression was reduced in cells. (**C**) Wound-healing assay of CRC cells with KLK10 knockdown monitored for 48-h with 24-h intervals. (**D**) Correlations between KLK10 expression and EMT markers (E-cadherin, N-cadherin, and Vimentin) using Spearman analysis. (**E**, **F**) The protein levels of E-cadherin, N-cadherin, and Vimentin were measured by western blotting after KLK10 knockdown in two CRC cell lines. Data are presented as mean ± SD. ^*^P < 0.05; ^**^P < 0.01; ^***^P < 0.001.

To determine the biological characteristics of KLK10, we performed GSVA enrichment analysis between the low- and high-expression groups. The results demonstrated that KLK10 was involved in cancer development and progression mechanisms, including the TP53, MAPK, and VEGF pathways, apoptosis pathway, chemokine pathway, and tumor metastasis pathway (Figure 10H), indicating that KLK10 may play an essential role in tumor growth and invasion. To validate our results further, specific siRNAs were transfected into HT29 and HCT116 cells, and the transfection efficiency of KLK10 siRNA was verified by qRT-PCR (Figure 11A). In terms of cell proliferation, the results of the CCK8 assay indicated that a reduction in KLK10 significantly suppressed the proliferation ability of HT29 and HCT116 cells (Figure 11B). Further, the results of wound-healing assays showed that the knockdown of KLK10 markedly restrained the healing of scratched wounds (Figure 11C). As we know, the epithelial-mesenchymal transition (EMT) is one of the key mechanisms of tumor metastasis and one of the main factors leading to the poor prognosis in patients. We conducted a correlation analysis and presented our findings in a heatmap (Figure 11D). The results indicated that the KLK10 expression was positively associated with the mesenchymal markers (e.g., N-cadherin and vimentin), while being negatively related to the epithelial markers (E-cadherin). In line with these outcomes, western blot analysis confirmed that the knockdown of KLK10 downregulated the protein level of N-cadherin and vimentin in two cell lines. On the contrary, reduced KLK10 expression markedly upregulated the protein level of E-cadherin (Figure 11E, 11F). Collectively, these results highlight the pro-tumor effects of KLK10 in CRC, in accordance with the results of bioinformatics analysis.

### KLK10 was significantly associated with *Fusobacterium nucleatum* (*F.n*) infection in CRC

**Figure 12:**
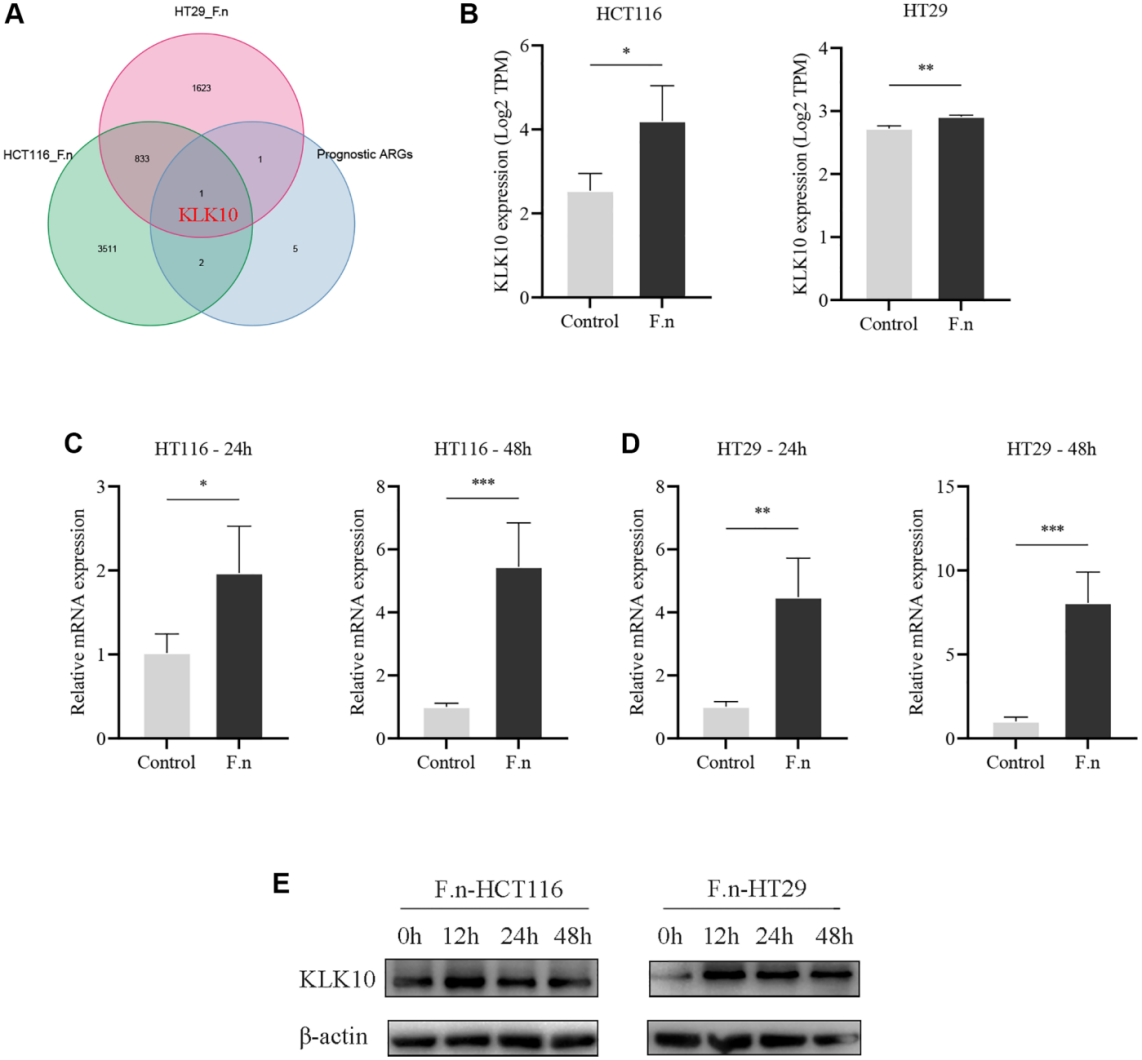
**CRC cells co-cultured with *F.n* were found to have significantly upregulated KLK10 expression.** (**A**) The Venn diagram demonstrating that KLK10 was shared by *F.n* infection-related DEGs and prognostic ARGs. (**B**) Microarray analysis of KLK10 expression between HCT116 cells and *F.n* infected HCT116 cells (left panel) and expression of KLK10 in HT29 cells incubated with or without *F.n* (right panel). (**C**, **D**) The KLK10 mRNA expression in CRC cell lines infected with or without *F.n* for 24 h and 48 h, respectively, was determined by qPCR. (**E**) Representative western blot for KLK10 protein and β-actin protein extracted from CRC cells infected with *F.n* for 0, 12, 24, and 48 h. ^*^P < 0.05; ^**^P < 0.01; ^***^P < 0.001.

The gut microbiota plays an important role in human health and disease. Recent studies have demonstrated that the presence of microbiota in various tumor tissues is not accidental, but a common phenomenon [61]. Therefore, the intratumoral microbiota is also considered to be part of the TME. *F.n* is a gram-negative anaerobe parasitic in the oral cavity, which has been found to promote CRC development through inhibits host anti-tumor immunity [62, 63]. Given the essential roles played by ARGs in the tumor immune microenvironment, we speculated that *F.n* dysregulation might affect the expression of ARGs and hence modulate local immune responses, in turn affecting immunotherapy. To determine the key ARGs associated with *F.n*, we performed whole genome microarray analysis of HCT116 cell lines infected with *F.n* for 48 h. In addition, the gene expression profile of the RNA-seq dataset (GSE90944) from HT29 cell lines infected with *F.n* was also obtained and further analyzed. Surprisingly, KLK10 was identified as the key downstream ARG of *F.n* (DEGs was set as an FDR < 0.05 and |log_2_ FC| ≥ 1) (Figure 12A). Moreover, the results showed that the expression of KLK10 was significantly upregulated by *F.n* in two cell lines (Figure 12B). Further, qPCR was performed to validate the expression of KLK10 in CRC cell lines infected with *F.n*. As shown in Figure 12C, 12D, the mRNA expression of KLK10 was significantly upregulated in CRC cells by *F.n* infection for 24 h and 48 h, respectively. These findings were consistent with the results of western blot analysis (Figure 12E). Overall, our findings suggest that *F.n* infection strongly induces KLK10 expression in CRC cell lines. As such, KLK10 could serve as a promising clinical indicator and therapeutic target for CRC patients infected with *F.n*.

## DISCUSSION

Evidence highlighting the critical role played by angiogenic factors in immune regulation and CRC progression is increasing [64–66]. Angiogenic factors excreted by CRC cells are known to stimulate endothelial cells to regulate angiogenic switches during CRC development [67]. In addition, angiogenic factors contribute to a pattern of impaired immune activation (immunosuppression) by activating suppressive immune cells or inhibiting immune effector cells, which can in turn stimulate angiogenesis and tumor progression [68, 69]. Notably, many researchers have shown that the indispensable relationship between angiogenesis and innate immunity and angiogenesis targeting has improved the immunotherapy efficacy and prognostic outcomes of tumor patients [70–73]. However, at present, there is a lack of bioinformatics analyses that demonstrate the holistic impact and TME cells infiltration features regulated by the combined role of multiple ARGs in CRC.

Herein, we demonstrated global alterations in ARGs at the transcriptional and genetic level. Most of them were elevated in advanced CRC patients (stage III–IV) and related to RFS. Next, we identified two distinct angiogenesis subgroups (Cluster A and B) based on 36 ARGs, and Cluster B was found to have more advanced clinicopathological characteristics and worse RFS compared to Cluster A. Furthermore, significant discrepancies were observed in TME infiltrations and biological functions between the two subgroups. Given the crucial roles played by gene mutations in the efficacy of immunotherapy, we further identified three gene_Clusters with different clinicopathological characteristics, immune cell abundance, and functional characterization based on the DEGs between the two angiogenesis subgroups. To quantify the angiogenesis subgroups, a prognostic ARG_score with robust predictive ability was constructed. The Cluster B and gene Cluster C with the poorest RFS had the greatest ARG_score among the two ARG subgroups and three gene subgroups. Patients with low- and high-ARG_score exhibited significantly distinct clinical outcomes, indicating that the ARG_score could predict poor clinical outcomes. Angiogenesis is confirmed that play a part in the aggressiveness of CRC [74, 75]. Interestingly, differences in mRNA transcriptomes between different angiogenesis subgroups were markedly enriched in cancer- and metastasis-related pathways, consistent with the existing conclusions.

The clinicopathological characteristics differed significantly between the low- and high-ARG_score groups. Multivariate Cox regression analysis showed that ARG_score could be used as an independent predictor of RFS in CRC patients. Its predictive robustness for 1-, 5-, and 10-year RFS were validated by ROCs. Moreover, we confirmed that ARG_score could be used for the prognosis stratification of CRC patients, implying that the ARG_score may have a reliable predictive capacity. Further, a quantitative nomogram was established to improve the performance and facilitate the use of the model by integrating the ARG_score and tumor stage. Recently, chemoresistance has become a major challenge, and the prognosis for patients with CRC can be very poor due to disease recurrence [76]. To address this, we investigated the sensitivity of patients in different ARG_score groups to different drugs. Our findings indicated that combination with targeting angiogenesis was conducive to ameliorating drug resistance and improving prognosis. In this context, TMB and MSI are considered as response predictors for tumor immunotherapy, including CRC [77, 78]. Our results revealed that there were significant differences between TMB and MSI between the low- and high-ARG_score groups. Furthermore, the high TMB and MSI-H groups have been previously demonstrated to be associated with a better prognosis for CRC patients [14, 79], consistent with our expected results. Results from clinical trials have demonstrated that anti-PD-1 antibodies can ultimately achieve a lasting complete response in CRC patients [80, 81]. In the present study, the expression of immune checkpoints was found to be significantly different in the high- and low-ARG_score groups. Thus, our results suggested that the ARG_score can be used to predict the efficacy of targeting angiogenesis therapy and immunotherapy, and in conjunction with targeted therapy, may be a significant adjustive strategy for CRC immunotherapy. Taken together, these findings indicate that the ARG_score can be used as a predictor of response to immunotherapy and clinical outcomes independent of the TMB, and may also be useful in assessing the MSI status of CRC patients effectively.

Evidence has increasingly shown that the TME plays a vital role in carcinogenesis, tumor progression, and drug resistance [82]. Currently, CRC patients continue to exhibit immunotherapy heterogeneity in their clinical outcomes, highlighting the important effects of the TME on CRC progression. The TME surrounding tumor cells is mainly composed of stromal cells and immune cells, such as tumor infiltrating immune cells, blood vessels, and tumor-associated fibroblasts [83]. Further, we evaluated the immune and stromal scores using the ESTIMATE algorithm and found that the high ARG_score groups significantly presented higher stromal scores than the low ARG_score groups, while the immune scores were poorer than those of the low ARG_score groups. These findings suggest that angiogenesis could be related to the involvement of the TME, thus regulating CRC tumorigenesis and progression. Granulocytes, lymphocytes, and macrophages are primary tumor-infiltrating immune cells elements of the TME, and have been proven to take part in many host immune pathways, including inflammatory responses mediated by the tumor to improve survival [84]. Tumor-associated macrophages are mainly categorized into M1 macrophages and M2 macrophages. M1 macrophages are characterized by anti-tumor function due to their ability to produce type I pro-inflammatory cytokines, while M2 macrophages contribute to the formation of immunosuppressive microenvironment and matrix remodeling, hence accelerating tumor growth [85, 86]. We noticed that the low ARG_score groups demonstrated favorable survival with more infiltrations of M1 macrophages, while increased infiltrations of M2 macrophages were observed in the high ARG_score groups with a worse prognosis. These findings are consistent with several previous studies. In addition, effector T cells, memory T cells, and T cell differentiation have been shown to play a crucial role in the immune defense of CRC, and higher densities of tumor-infiltrating T cells in CRC tissues indicate a good prognosis [87, 88]. We demonstrated that the low ARG_score groups showed a higher infiltration of activated memory CD4+, CD8 + T, and follicular helper T cells, indicating that they have a positive effect on CRC prognosis. Recent studies have revealed that the infiltration of Tregs can suppress anti-cancer immunoreactivity, and hence favor tumor growth [89]. This is consistent with our finding of a higher Treg infiltration in the TME of patients with a high ARG_score.

Kallikrein-related peptidase 10 (KLK10) is a member of the KLKs family with tryptic or chymotryptic activity [90, 91]. Several studies have found that CRC patients with high KLK10 expression have a poorer prognosis [92, 93]. In this study, we confirmed that KLK10 expression was significantly upregulated at the mRNA level in CRC tissues compared to adjacent normal tissues. Prognosis analysis showed that higher levels of KLK10 expression in patients was significantly associated with a shorter RFS, which was consistent with the results of previously published studies. However, the roles of KLK10 in CRC development, immune infiltration, and immunotherapy response remain poorly understood. Functional enrichment analysis showed that higher KLK10 expression was involved in tumor cells adhesion, invasion, metastasis, and immune cell infiltration, which highlighted the potential mining value of KLK10 in CRC progression and the tumor immune microenvironment. Further *in vitro* experiments were performed to confirm that the knockdown of KLK10 in CRC cells markedly decreased in cell viability and proliferation. In addition, this study was the first to suggest that the downregulation of KLK10 suppressed the migration and invasion of CRC cells through the inhibition of the EMT. Recently, KLK10 was identified as a potential target for immunotherapy based on the immunopeptidome analysis of ovarian cancer antigens [94]. The results of different algorithms in immune cell infiltration showed that high KLK10 expression may be conducive to an immune hot type. Moreover, the TIDE analysis implied that patients with high KLK10 expression were more susceptible to tumor immune escape, in accordance with previous studies. *F.n* has been confirmed to play an important role in the occurrence and development of CRC. Interestingly, we explored the sequencing results obtained after CRC cells were infected with *F.n*, and discovered that KLK10 expression was significantly upregulated. *F.n* may affect CRC cell proliferation, invasion, metastasis, and drug resistance via the upregulation of KLK10, and hence change the clinical outcomes of patients. At present, there are no studies on the impact of *F.n* on the survival and development of CRC regulated by the expression of ARGs. However, further systematic molecular experiments may elucidate novel pathogenic pathways and therapeutic targets.

This study has some limitations. Namely, our results were obtained by analyzing public databases (TCGA and GEO), and future studies will need to incorporate the latest clinical samples for further verification. Furthermore, multicenter clinical immunotherapy cohorts should be performed to confirm the robustness and consistency of the ARG_score in predicting prognosis and immunotherapy efficacy, which will be the emphasis of future research. Lastly, complementary *in vivo* and *in vitro* studies will be needed to verify our findings. Our laboratory is further investigating the important links between these factors.

## CONCLUSIONS

In this study, a novel angiogenesis-related molecular subtype based on ARGs in CRC was identified, providing a comprehensive assessment of the heterogeneity and complexity of TME. We also identified the therapeutic orientation of ARGs in targeting therapy and immunotherapy. Although these findings highlight the vital clinical implications of ARGs, future studies in multi-centers on a larger scale are essential. This study is the first to describe the potential contribution of *F.n* to the transcriptional changes in ARGs, and provides specific directions for further research. The results presented here suggest that ARGs could be used as an effective biomarker to predict prognosis and immunotherapy response in patients with CRC, highlighting their potential involvement in tumor development in *F.n*-infected CRC patients. Further *in vitro* or *in vivo* experiments should be conducted to verify the relationships between these factors.

## Supplementary Materials

Supplementary Figures

Supplementary Tables 1, 2 and 4

Supplementary Tables 3, 5 and 6
