# Supplementary material for: Construction and validation of a novel angiogenesis pattern to predict prognosis and immunotherapy efficacy in colorectal cancer
Source: Aging (Albany NY). 2023 Nov 7;15(21):12413–50. doi: 10.18632/aging.205189 (PMC10683615; doi:10.18632/aging.205189)
Supplement: Supplementary Figures [file aging-15-205189-s001.pdf]

SUPPLEMENTARY FIGURES

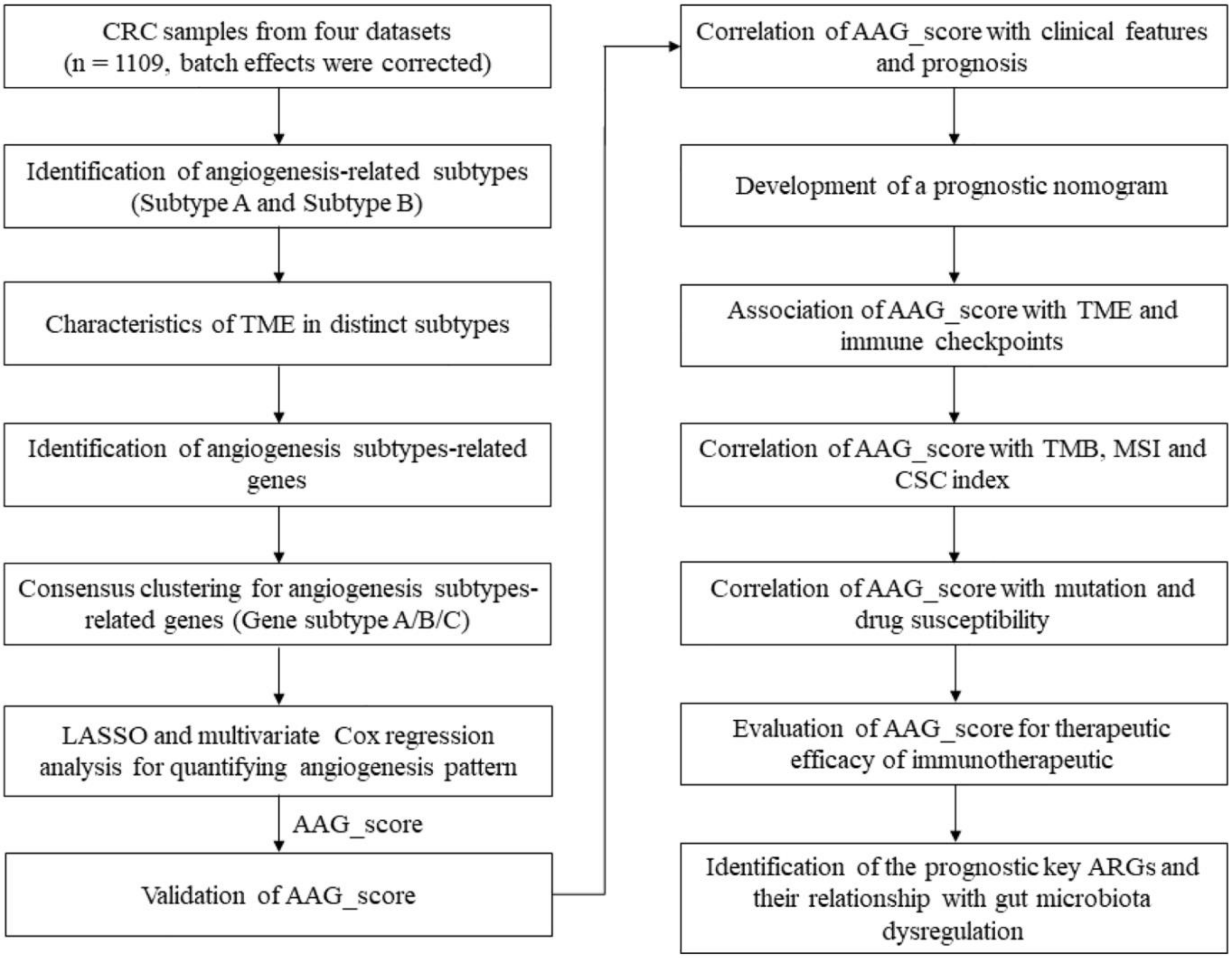

Supplementary Figure 1. The specific workflow graph of data analysis.

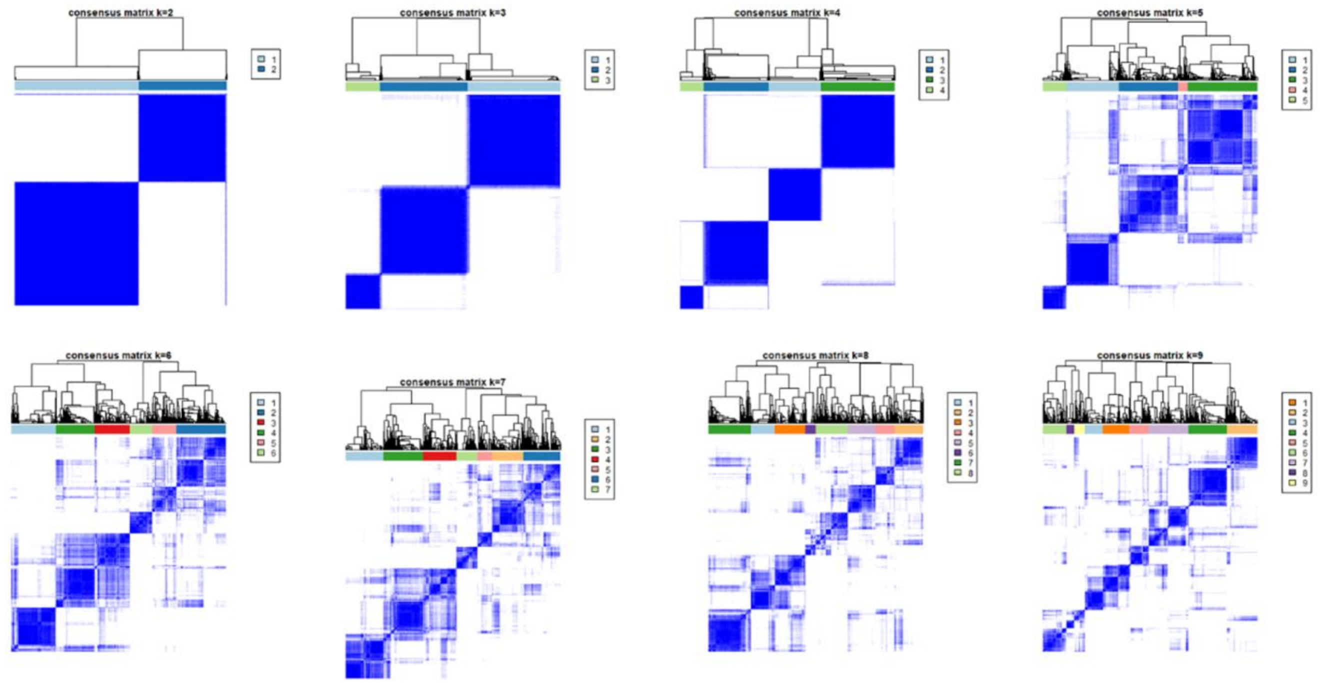

Supplementary Figure 2. Unsupervised clustering of gene subtypes based on DEGs and Consensus matrix heatmaps for k = 2–9.

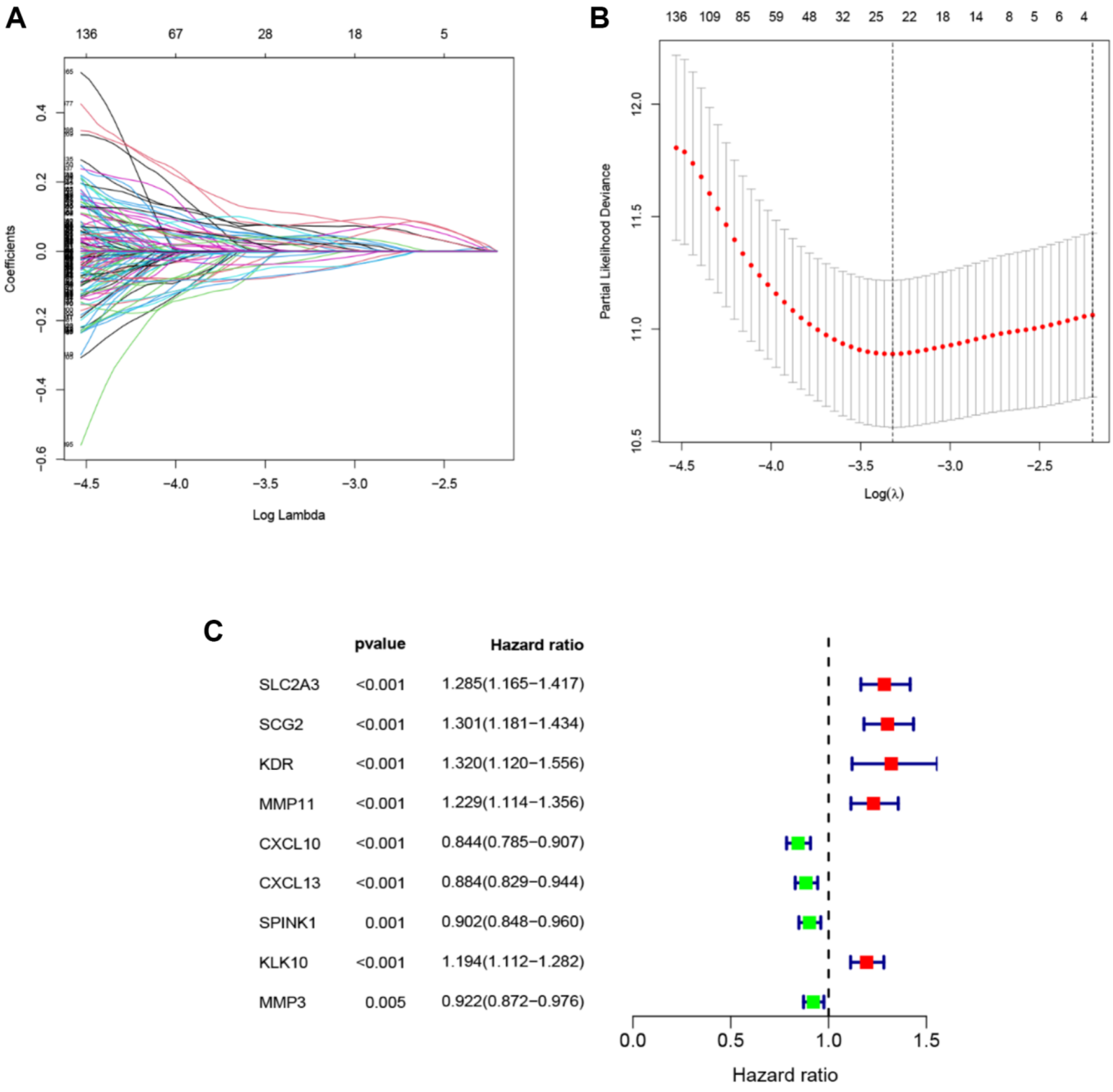

Supplementary Figure 3. Identifying representative candidate prognostic genes. (**A**, **B**) The LASSO regression analysis and partial likelihood deviance on the prognostic genes. (**C**) Forest plot of multivariate cox regression analysis for prognostic genes.

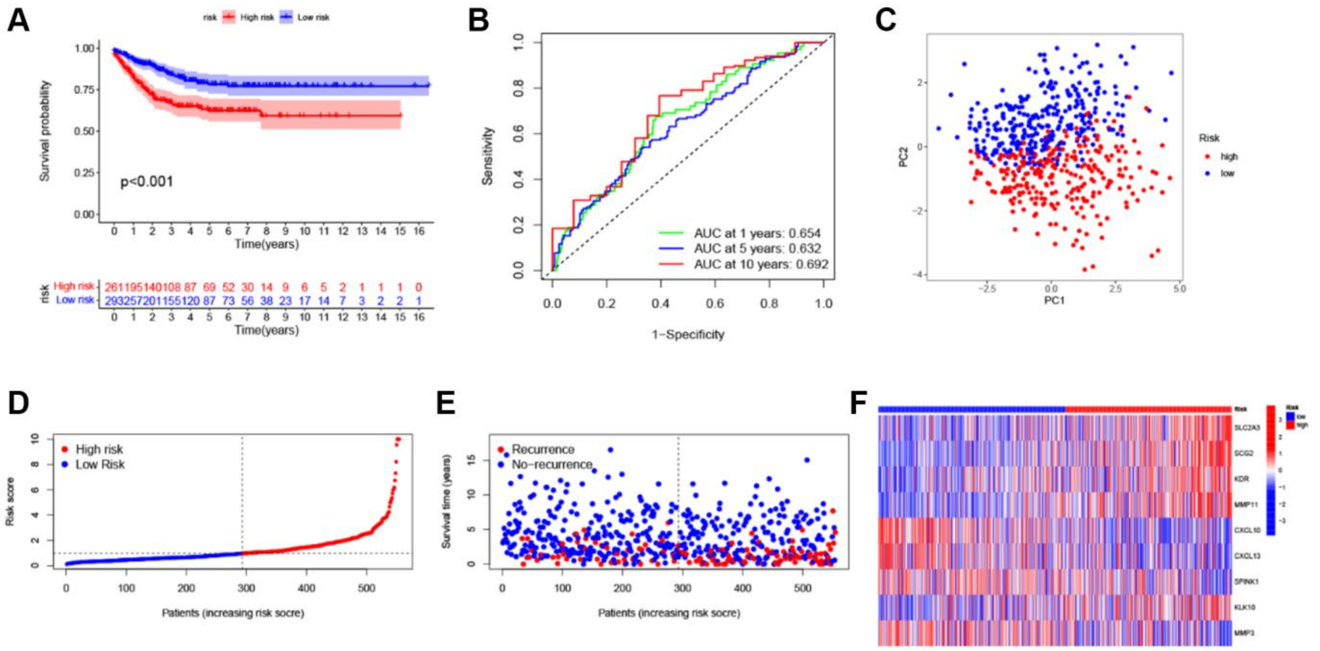

Supplementary Figure 4. Validation of ARG_score in testing cohort. (**A**) K-M analysis of the RFS between the two ARG_score groups. (**B**) ROC curves to predict the sensitivity and specificity of 1-, 5-, and 10-year survival according to the ARG_score. (**C**) The PCA analysis demonstrated that the patients in the different ARG_score groups were distributed in two directions. (**D**, **E**) The ranked dot plot indicates the ARG_score distribution and scatter plot presenting the patients' survival status. (**F**) Expression patterns of 9 selected prognostic genes in high- and low-ARG_score groups.

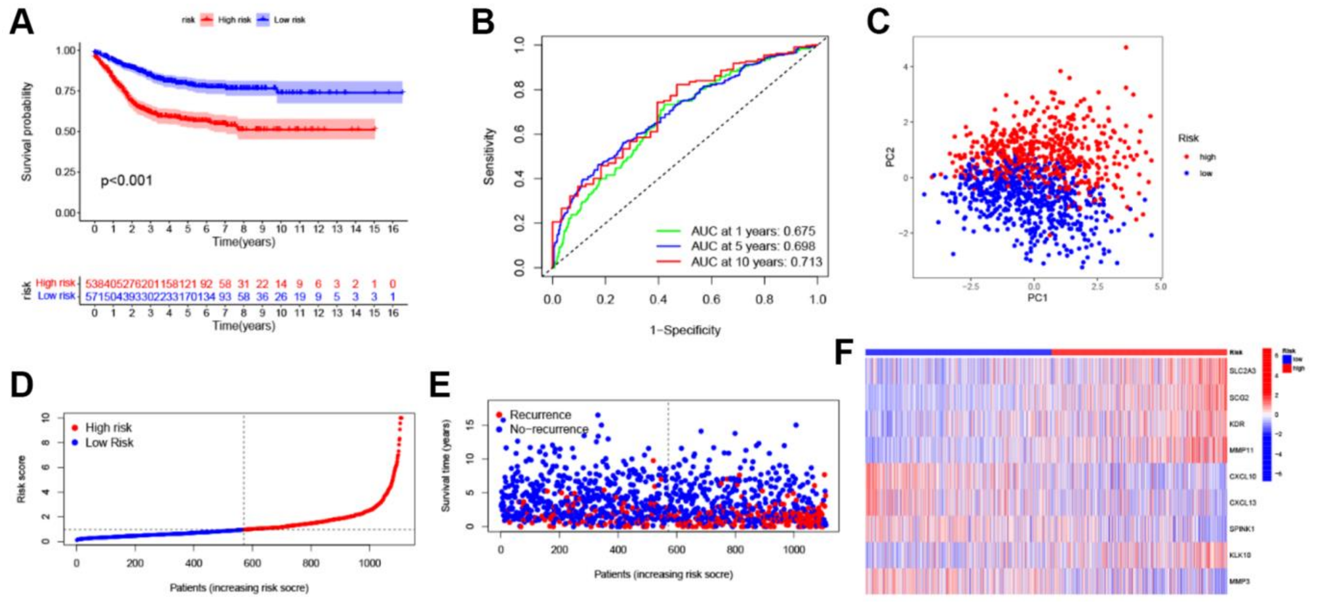

Supplementary Figure 5. Validation of ARG_score in entire cohort. (**A**) K-M analysis of the RFS between the two ARG_score groups. (**B**) ROC curves to predict the sensitivity and specificity of 1-, 5-, and 10-year survival according to the ARG_score. (**C**) The PCA analysis demonstrated that the patients in the different ARG_score groups were distributed in two directions. (**D**, **E**) The ranked dot plot indicates the ARG_score distribution and scatter plot presenting the patients' survival status. (**F**) Expression patterns of 9 selected prognostic genes in high- and low-ARG_score groups.

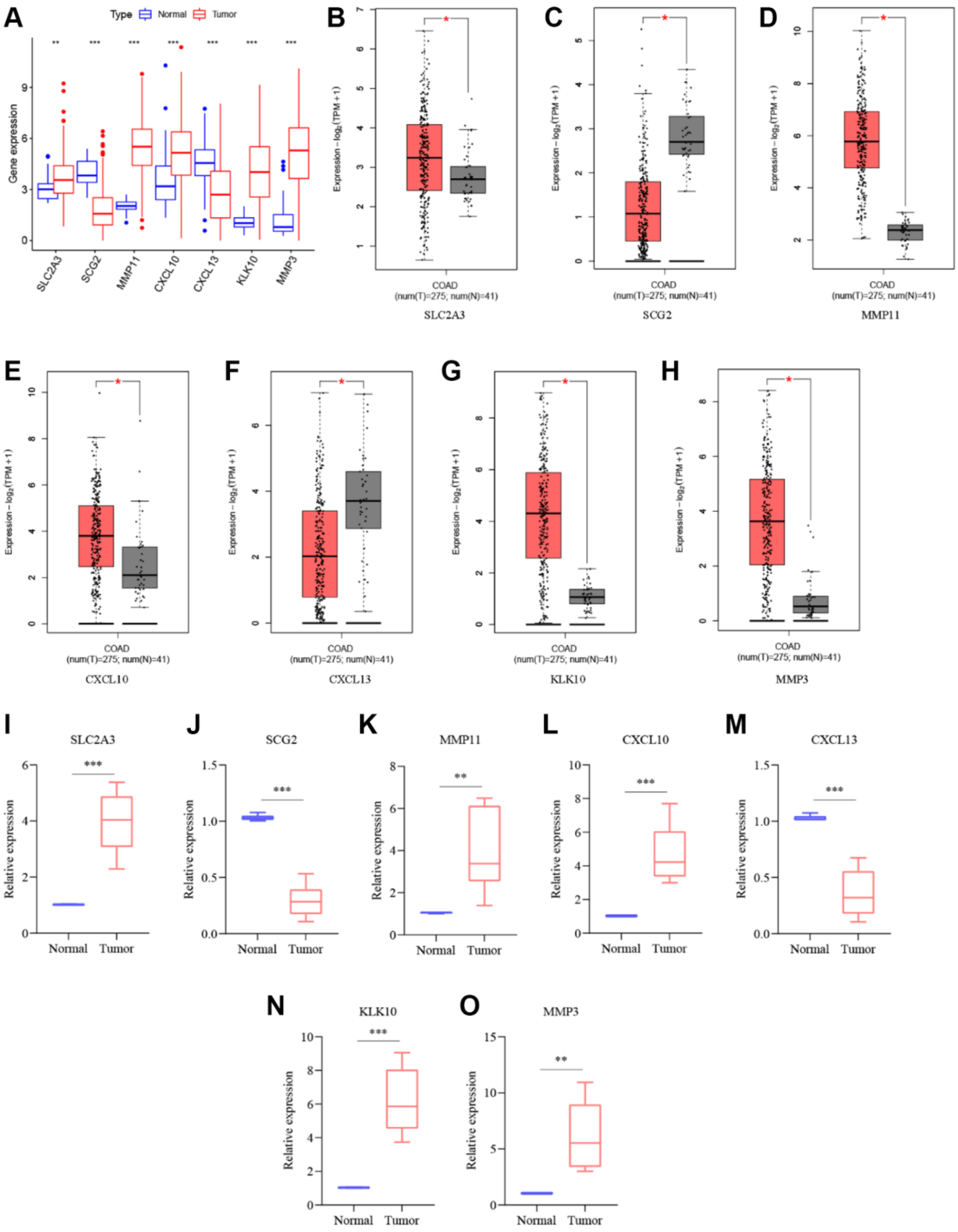

Supplementary Figure 6. Expression levels of 9 selected angiogenesis-related prognostic genes in CRC tissues and corresponding normal tissues. (**A**) Expression of 9 prognostic genes in CRC tissues and normal human colon tissues (TCGA cohort). (**B**–**H**) Expression of 9 prognostic genes in CRC tissues and normal human colon tissues (GEPIA 2 cohort). (**I**–**O**) Expression levels of 9 prognostic genes in CRC tissues and corresponding normal tissues by RT-PCR. $^{*}P < 0.05$; $^{**}P < 0.01$; $^{***}P < 0.001$.

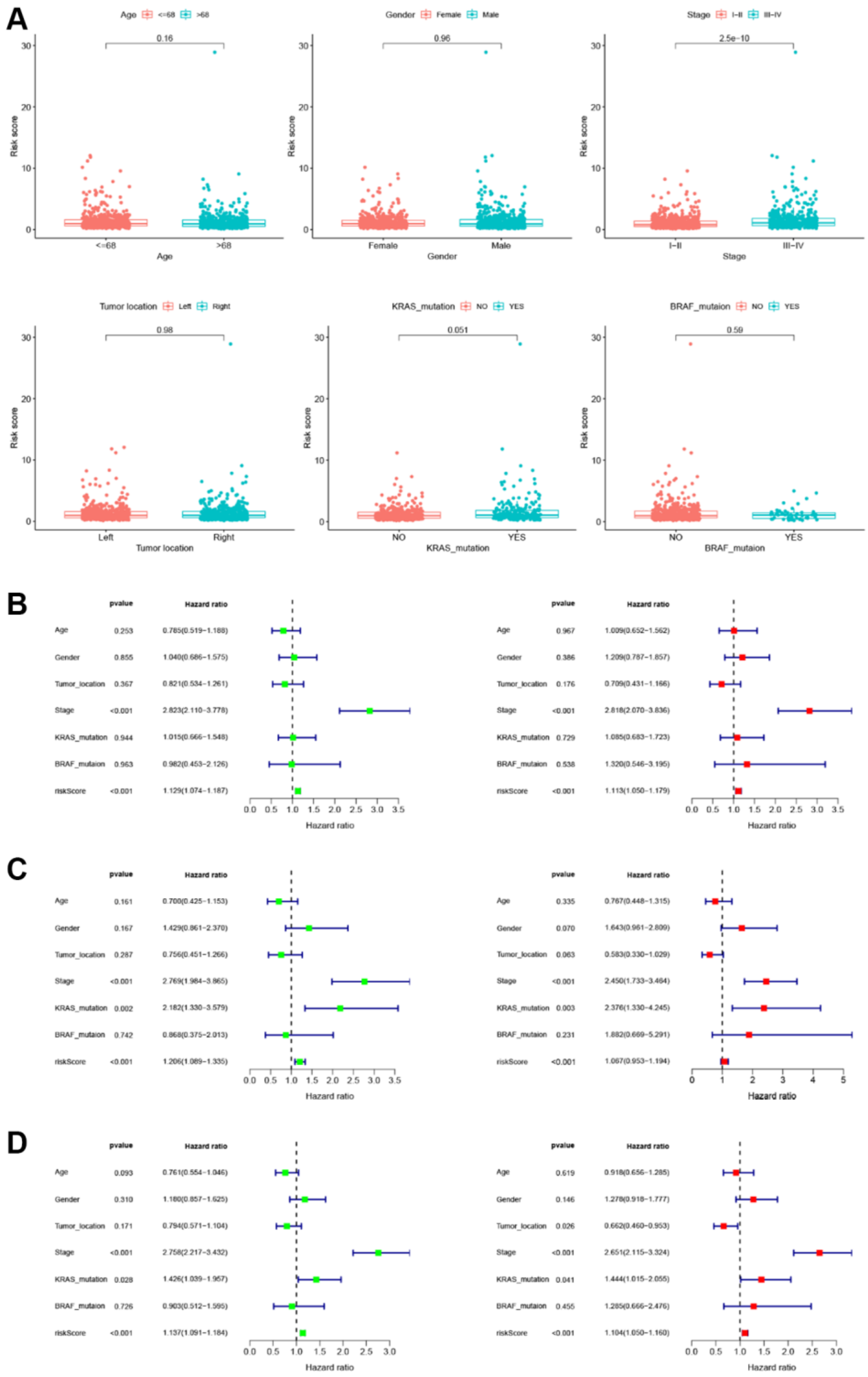

Supplementary Figure 7. The correlation and independent prognosis analysis of ARG_score and clinicopathological variables in CRC. (**A**) The correlation between the ARG_score and clinical characteristics, including age, gender, TNM stage, tumor location, KRAS mutation status, and BRAF mutation status. (**B**) Univariate and multivariate analyses showed the prognostic value of the ARG_score in the training cohort. (**C**) Univariate and multivariate analyses showed the prognostic value of the ARG_score in the testing cohort. (**D**) Univariate and multivariate analyses showed the prognostic value of the ARG_score in the entire cohort.

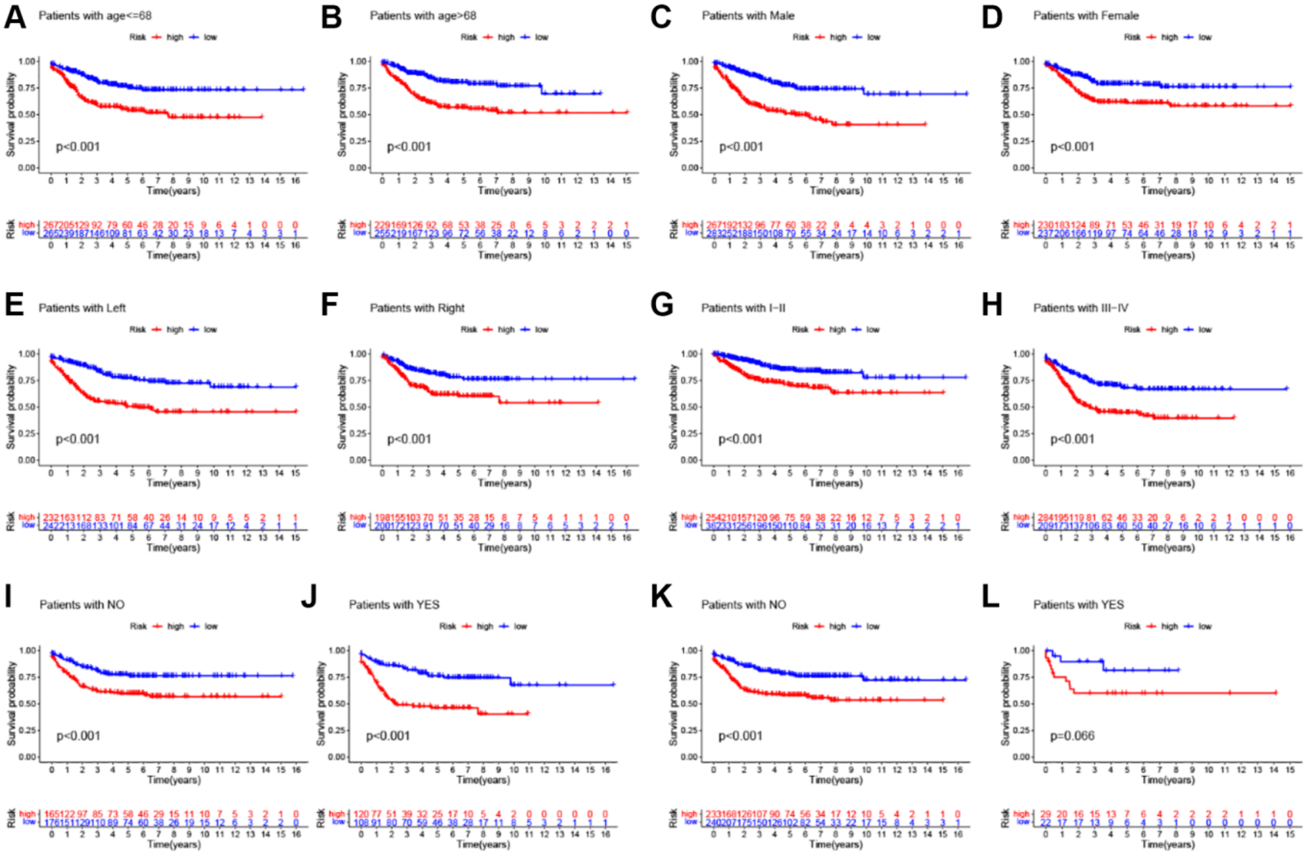

Supplementary Figure 8. Stratification analysis of the ARG_score in CRC. (**A**, **B**) Age (age ≤ 68 and age > 68 years old). (**C**, **D**) Gender (female and male). (**E**, **F**) Tumor location (left-side or right-side). (**G**, **H**) Tumor stage (I–II or III–IV). (**I**, **J**) KRAS mutation (yes and no). (**K**, **L**) BRAF mutation (yes and no).
