# Supplementary material for: Construction and validation of a novel angiogenesis pattern to predict prognosis and immunotherapy efficacy in colorectal cancer
Source: Aging (Albany NY). 2023 Nov 7;15(21):12413–50. doi: 10.18632/aging.205189 (PMC10683615; doi:10.18632/aging.205189)
Supplement: Supplementary Tables 1, 2 and 4 [file aging-15-205189-s002.pdf]

SUPPLEMENTARY TABLES

Please browse Full Text version to see the data of Supplementary Tables 3, 5 and 6.

Supplementary Table 1. Summary of 36 recognized ARGs.

Gene	Type
VCAN	Angiogenesis
POSTN	Angiogenesis
FSTL1	Angiogenesis
LRPAP1	Angiogenesis
STC1	Angiogenesis
LPL	Angiogenesis
VEGFA	Angiogenesis
PF4	Angiogenesis
THBD	Angiogenesis
FGFR1	Angiogenesis
TNFRSF21	Angiogenesis
CCND2	Angiogenesis
COL5A2	Angiogenesis
ITGAV	Angiogenesis
SERPINA5	Angiogenesis
KCNJ8	Angiogenesis
APP	Angiogenesis
JAG1	Angiogenesis
COL3A1	Angiogenesis
SPP1	Angiogenesis
NRP1	Angiogenesis
OLR1	Angiogenesis
PDGFA	Angiogenesis
PTK2	Angiogenesis
SLCO2A1	Angiogenesis
PGLYRP1	Angiogenesis
VAV2	Angiogenesis
S100A4	Angiogenesis
MSX1	Angiogenesis
VTN	Angiogenesis
TIMP1	Angiogenesis
APOH	Angiogenesis
PRG2	Angiogenesis
JAG2	Angiogenesis
LUM	Angiogenesis
CXCL6	Angiogenesis

Supplementary Table 2. The primer sequences for qRT-PCR.

Gene	Primer Sequence
CXCL13	F: 5′-GGTCAGCAGCCTCTCTCCAGTC-3′
	R: 5′-TTGAATTCGATCAATGAAGCGT-3′
SLC2A3	F: 5′-GAAGAGGAGAATGCTAAG-3′
	R: 5′-CAATGGAAATGATGATGG-3′
CXCL10	F: 5′-GTGGCATTCAAGGAGTACCTC-3′
	R: 5′-GCCTTCGATTCTGGATTCAGACA-3′
KDR	F: 5′-CTACTGATTTTTGCCCTTGTTC-3′
	R: 5′-TAGTCATTGTTCCCAGCATTTC-3′
KLK10	F: 5′-TCTACCCTGGCGTGGTCACC-3′
	R: 5′-GCAGAGCCACAGGGGTAAACAC-3′
MMP11	F: 5′-AAGAGGTTCGTGCTTTCTGG-3′
	R: 5′-CCATGGGAACCGAAGGAT-3′
MMP3	F: 5′-CCTGCTTTGTCCTTTGATGC-3′
	R: 5′-TGAGTCAATCCCTGGAAAGTC-3′
SCG2	F: 5′-ACCAGACCTCAGGTTGGAAAA-3′
	R: 5′-ACCAGACCTCAGGTTGGAAAA-3′
SPINK1	F: 5′-TGTCTGTGGGACTGATGGAA-3′
	R: 5′-GCCCAGATTTTTGAATGAGG-3′
β-actin	F: 5′-GCATGGAGTCCTGTGGCAT-3′
	R: 5′-CTAGAAGCATTTGCGGTGG-3′
siKLK10#1	5′-CCTCCACACCTCTAAACATCTC-3′
siKLK10#2	5′-TTGTTGTACTTCACTCTCCGG-3′
siKLK10-NC	5′-UUCUCCGAACGUGUCACGUTT-3′

Supplementary Table 3. Clinical information of 1109 colorectal cancer patients.

Supplementary Table 4. The prognostic values of 36 ARGs in patients with CRC.

id	HR	HR.95L	HR.95H	*P*-value	km
VCAN	1.224994274	1.11397604	1.347076523	2.83E-05	1.19E-06
POSTN	1.181163635	1.081386881	1.29014653	0.000217649	1.66E-06
FSTL1	1.273841191	1.118843592	1.450311189	0.0002558	5.61E-06
LRPAP1	0.67653805	0.510799323	0.896053914	0.006421073	0.000186841
STC1	1.201646156	1.066394221	1.354052241	0.002568988	0.000953075
LPL	1.19409106	1.094679196	1.302530881	6.34E-05	2.38E-05
VEGFA	1.466477431	1.219837129	1.762986225	4.60E-05	2.23E-06
PF4	0.947950341	0.865382536	1.038396098	0.250296529	0.048438335
THBD	1.222489097	1.040031194	1.436956507	0.01485467	0.005430823
FGFR1	1.31569863	1.108971297	1.56096275	0.001655545	0.000456971
TNFRSF21	1.165541157	0.937967838	1.448329178	0.166925139	0.008317478
CCND2	0.905160617	0.809083578	1.012646611	0.081779674	0.000854507
COL5A2	1.237035349	1.121895833	1.363991568	1.98E-05	5.01E-07

ITGAV	1.249982819	1.078549708	1.448664847	0.00303005	0.003795799
SERPINA5	1.197708641	1.027155793	1.396580732	0.021345418	0.001044201
KCNJ8	1.389973359	1.213983433	1.591476364	1.87E-06	1.27E-08
APP	1.062376145	0.833601656	1.353935739	0.624820263	0.078671415
JAG1	1.016142325	0.838207208	1.231849612	0.870487009	0.030863117
COL3A1	1.241335579	1.121609276	1.373842079	2.94E-05	1.20E-07
SPP1	1.116360262	1.047764384	1.189447029	0.000668835	7.87E-05
NRP1	1.317014905	1.13423922	1.529243769	0.000303391	2.38E-05
OLR1	1.17399583	1.078246613	1.278247659	0.000219433	0.00037697
PDGFA	0.913533642	0.77915275	1.071091278	0.265285904	0.110789116
PTK2	1.307826879	1.020229861	1.676495868	0.034172484	0.009839317
SLCO2A1	1.076135281	0.939249225	1.232971093	0.290480775	0.053553315
PGLYRP1	0.916923323	0.703850283	1.194498888	0.52036185	0.141275291
VAV2	1.139619561	0.966985174	1.343074102	0.118902964	0.008376223
S100A4	1.198276931	1.084160975	1.324404436	0.00039635	7.44E-05
MSX1	0.959960251	0.833859773	1.105130278	0.569546779	0.069710173
TIMP1	1.390530524	1.186145976	1.630132527	4.81E-05	2.03E-05
APOH	0.978210944	0.820736312	1.165900225	0.805686095	0.116002365
PRG2	0.982731271	0.71948208	1.342299939	0.912807991	0.133462913
JAG2	1.088750856	0.937652326	1.264198246	0.26465296	0.009779996
LUM	1.140747631	1.03752703	1.254237354	0.006503175	0.001225002
CXCL6	0.942957149	0.863324081	1.029935575	0.191984201	0.002126871

Supplementary Table 5. Spearman correlation analysis of the 36 ARGs.

Supplementary Table 6. Prognostic analysis of 620 subtype-related genes using a univariate Cox regression model.
